# The Coevolution of Biomolecules and Prebiotic Information Systems in the Origin of Life: A Visualization Model for Assembling the First Gene

**DOI:** 10.3390/life12060834

**Published:** 2022-06-02

**Authors:** Sankar Chatterjee, Surya Yadav

**Affiliations:** 1 Department of Geosciences, Museum of Texas Tech University, Box 43191, 3301 4th Street, Lubbock, TX 79409, USA; 2 Rawls College of Business, Texas Tech University, Box 42102, 703 Flint Avenue, Lubbock, TX 79409, USA; surya.yadav@ttu.edu

**Keywords:** coevolution of biomolecules and information systems, peptide/RNA world, hydrothermal crater lakes, prebiotic information systems, analog information, hybrid information, digital information, memory transfer and memory bank, the genetic code, AnyLogic visualization of encoding mRNAs by tRNAs

## Abstract

Prebiotic information systems exist in three forms: analog, hybrid, and digital. The Analog Information System (AIS), manifested early in abiogenesis, was expressed in the chiral selection, nucleotide formation, self-assembly, polymerization, encapsulation of polymers, and division of protocells. It created noncoding RNAs by polymerizing nucleotides that gave rise to the Hybrid Information System (HIS). The HIS employed different species of noncoding RNAs, such as ribozymes, pre-tRNA and tRNA, ribosomes, and functional enzymes, including bridge peptides, pre-aaRS, and aaRS (aminoacyl-tRNA synthetase). Some of these hybrid components build the translation machinery step-by-step. The HIS ushered in the Digital Information System (DIS), where tRNA molecules become molecular architects for designing mRNAs step-by-step, employing their two distinct genetic codes. First, they created codons of mRNA by the base pair interaction (anticodon–codon mapping). Secondly, each charged tRNA transferred its amino acid information to the corresponding codon (codon–amino acid mapping), facilitated by an aaRS enzyme. With the advent of encoded mRNA molecules, the first genes emerged before DNA. With the genetic memory residing in the digital sequences of mRNA, a mapping mechanism was developed between each codon and its cognate amino acid. As more and more codons ‘remembered’ their respective amino acids, this mapping system developed the genetic code in their memory bank. We compared three kinds of biological information systems with similar types of human-made computer systems.

## 1. Introduction

One of the most enduring mysteries of modern science has been the origin of life on Earth and its information systems. The clues for the origin of life come from astrobiology, the early Earth, biochemistry, molecular biology, and laboratory experiments. Because information permeates life, any study of abiogenesis must address the origin of biological information systems as well. Information is one key property, perhaps the key property, that separates life from nonlife. It is the logic of life that makes a living system more organized, ordered, and complex. The way the information flows through and between biomolecules and cells is unique in nature [1]. Earth’s biological and informational evolution are intertwined and inseparable. Life and its information systems form a closely coupled entity, influencing each other in a complex feedback loop. Life is an information processing system that can store and process information necessary for its growth, metabolism, and self-reproduction. Life transmits heritable information to its progeny and undergoes Darwinian evolution. This is how life begets life and creates biodiversity. The information does not change whether it is encoded in nucleic acids or proteins: information is substrate independent. The concept of information is central to a meaningful description of biological processes, but its status as a physical entity remains elusive. Darwinian evolution tends to lead to an increase in information content and a decrease in randomness during abiogenesis. 

It is generally believed that two kinds of biological information, analog and digital, emerged about four billion years ago during abiogenesis [1,2,3]. De Duve [4] viewed pathways of life as both determinate and directional, where the vector of evolution lies in the structural, informational, and catalytic molecules. It is well-known in biochemistry that biomolecules are very sensitive to the changes in their environment—changes in pressure, temperature, pH concentration, ATP concentration, molecular count, etc. These elements are in constant flux, enabling and forcing biomolecules to be a dynamic and flexible system to adapt and respond quickly to the changes in the environment. Biomolecules have a reconfigurable internal structure that enables them to change in the best way to face the environment and solve the problem. They must also deal with limited resources and time. Analog computing is better suited for such a situation. It requires fewer parts, fewer resources, less energy, and less time than digital computing. Therefore, biomolecules—large and small—are analog machines with their own embedded analog information system. They perform analog computing. It is very instructive to understand the nature of a molecular analog information system. An analog information system’s internal structure is not fixed like a digital information system. Instead, an analog information system’s internal structure is reconfigurable and solves the problem (or situation) by changing its structure in a suitable way [5]. 

Each molecular unit has its own information and information system, meaning that ‘the information comes from within’. In other words, the molecular units may receive signals from the environment, but they contain their own information to process the signal. Each molecular unit performs its function using its own structure, information, and the information-function interdependency rules [6]. We assert that information contained and used by various molecular units is in the form of four major categories—time, space, control, and energy. The time information consists of temporal elements such as rate, clock, etc. The space information consists of spatial elements such as pattern, proximity, attractiveness, sequence, etc. The control information includes signal and regulatory elements. The energy information includes potential energy, charge differences, etc. The molecular units use, consume, and produce this information. 

Molecular information systems began early in the interstellar medium in the building blocks of life, which were delivered to young Earth by meteorites during the heavy bombardment period. Analog information systems appeared first in abiogenesis, followed by digital information systems. These two information systems, operating separately and in close cooperation, streamlined the prebiotic synthesis from chaotic molecular assemblages and provided directionality to the flow of information. 

A Digital Information System (DIS) includes the genetic information built slowly by the coded sequences of nucleotides in mRNA in the peptides/RNA world. It is a latecomer in abiogenesis. Digital information is discrete and is encoded in linear sequences of nucleotides in mRNA and later in DNA. The sequence of nucleotides in mRNA and DNA determines the information content of the molecules. Digital information processing in translation, genetic code, and transcription are familiar to us. Less appreciated are the analog aspects of information processing. 

The dichotomy between analog and digital information is not clear-cut. We show that between analog and digital, there is a transitional information stage, which we call the Hybrid Information System. The identification of these three systems helps us to document the coevolution of biomolecules and information systems during abiogenesis. These new approaches to prebiotic information systems are necessary for understanding the origin of life. 

Living things collect and store information from the environment for survival. They adapt to their environment by using the information to harvest energy and evade equilibrium. Life is characterized and sustained by several information-rich biological processes that govern cellular functions and significantly contribute to its overall complexity. Information is an important prerequisite for the onset of life. Prebiotic information would undoubtedly have been much simpler and was built incrementally over time. 

Yockey [7] differentiated the processes of analog and digital information systems. Analog information is spontaneous, blended, and three-dimensional; components come from within the molecules. In contrast, digital information is linear, sequential, segregated, and guided by coding rules. It is more robust and efficient to transmit information in digital form than in analog form. The digital code is inscribed in a template (mRNA or DNA) that provides the order in which the product (protein) is assembled. That order specifies biological specificity. Linear, digital, and specific properties do not exist in analog form. In digital information, both genes and proteins are manufactured or are artifacts produced by molecular machines, the former by the transcription machine, the latter by the translation machine [8]. A bilingual translation machine—a ribosome—orchestrates the translation of mRNA language to protein language. Life depends upon the interplay of both digital and analog coding, known as code-duality. The analog reaction, on the other hand, is entirely in monolingual chemical language. The demarcation between nonlife and life is that the former is made of spontaneous objects, whereas the latter is made of manufactured objects or artifacts. Life is information plus meaning. Digital information provides both the data and the meaning. The meaning involves two processes: (1) to manufacture proteins; (2) to perpetuate life. All life on Earth is possible because of a discrete digital mechanism of preservation and replication. Some of the information is encoded in the genes and passed on from one generation to the next.

We use metaphors such as analog and digital, software and hardware, nanobots, and computers, to compare life’s information systems with human-made machines. This comparison is subtle if we understand the limitations of these metaphors. For example, digital information is like a ‘program’, and an analog system is like a ‘computer’ to run the program of life. However, a cell is more complicated, reliable, and versatile than a supercomputer. As we trace the biochemical pathways for the origin of life, we see a continuum from the most basic and primitive forms of information to the most sophisticated forms of information in evolutionary terms.

Any modern complex computer operating system is partly digital and partly analog, and any living organism is an even more complex hybrid of digital and analog components. The concepts of analog and digital are far too narrow to encompass the subtleties of living cells. The analog vs. digital dichotomy here is more of an analogy than a precise description. However, these concepts provide working tools to investigate the origin of information systems in life. Here we describe a third possible form that is neither completely analog nor completely digital but a Hybrid Information System (HIS) that bridges the gap between the Analog Information System (AIS) and the Digital Information System (DIS).

Life depends not only on the flow of energy but also on the flow of information. A living system not only stores information, but it processes and uses the information to self-maintain and perpetuate itself. Living is an information processing system in which memory is maintained in analog, hybrid, and digital forms. A living system must store information and process and use it [9].

Information has been emerging and propagating itself in life for billions of years on Earth. Evolution is an information-generating and transmitting process. It creates information in a hierarchical structure and involves constraints, specializations, and symbiotic relationships. The origin of life has produced organic molecules of increasing size and complexity in collaboration with information systems through time. How can a living system emerge from a chaotic assemblage of space molecules in the hydrothermal vent environment? What rule might have guided the prebiotic synthesis? The rule of life is its information systems. It reduces the number of possible prebiotic interactions in the crater vent environment and compresses the evolutionary goal of reproduction [10]. It provides the directionality during abiogenesis. A computational view of life also could explain informational hierarchies as they exist across multiple functional spaces and times in the prebiotic synthesis. In this paper, we discuss the coevolution of biomolecules and information systems during the origin of life. Here we try to answer some fundamental questions about biological information: What is information? Where does it come from? Does it have any causality?

## 2. Objectives

The biological information systems had attracted the attention of early pioneers in information systems such as John von Neumann, Alan Turing, Claude Shannon, Norbert Wiener, and many others in the 1950s and 1960s. They made major contributions to our understanding of biological information systems. We want to revive this information-centric view in the origin of life study. 

In this paper, we propose four novel ideas of prebiotic information systems central to the origin of life. These are (1) recognition of hybrid information system in the origin of life, (2) the coevolution of biomolecules and prebiotic information systems, (3) the origin of the first gene before DNA, and (4) the comparison of prebiotic information systems with the information systems of the human-made machines. We followed a theoretical approach guided by the likely biochemical pathways because these events, which happened four billion years ago in the prebiotic world, were lost in modern cellular functions, which are difficult to prove with experiments in the laboratory.

### 2.1. Recognition of Hybrid Information Systems

Although analog and digital information systems are well-known in the literature, we identified a transitional form, Hybrid Information Systems (HIS), composed of noncoding RNAs. HIS gave rise to major components of the translation machine, such as tRNA (transfer RNA), aaRS (aminoacyl-tRNA synthetase), and ribosomes. These components originated from ribozymes. 

### 2.2. The Coevolution of Biomolecules and Prebiotic Information Systems

This is the main theme of the paper and is discussed extensively in the text. Information may hold the key to understanding the mystery of life’s origin. However, information’s status as a physical entity remains elusive. We identified three kinds of information systems in biomolecules—analog, hybrid, and digital—that emerged in succession. We show the coevolution of these tripartite information systems with life’s molecules during prebiotic synthesis.

### 2.3. The Emergence of the First Gene

A codon is a sequence of mRNA that corresponds with a specific amino acid or stop signal during protein synthesis. In living cells, a gene or mRNA molecule is formed from the transcription of DNA, but the origin of the gene was poorly understood before the advent of DNA. In the peptide/RNA world, an mRNA molecule encodes the information of amino acids for making a specific protein. Here, we offer a plausible biochemical pathway for the evolution of the first gene step-by-step by tRNA and the tRNA anticodon in the peptide/RNA world. Without mRNA, no life supported by genetic coding could evolve. Codons and amino acids do not recognize directly but use tRNAs for information content. How did an ancestral codon ‘remember’ its association with a specific amino acid? We propose a model of memory transfer from tRNA to mRNA to build codon–amino acid mapping. We see the memory transfer during the reproduction of the first cells from parents to daughters. 

Walker [11] discussed ‘biological memory’ in the context of an information system as a mapping mechanism—a relationship between input and the resulting output of an event. In some cases, the input of the information carrier disappears automatically with the output of an event. For example, when translated into protein, mRNA is destroyed and recycled. DNA, on the other hand, is an information storage system, and it remains intact after transcription. DNA is considered a permanent memory mapping of protein structure. It relegates mRNA for protein building, where the recipe of a specific protein is transcripted in the codon sequences of mRNA or a gene. 

Digital or genetic memory flourished during the buildup of mRNAs, translation, and genetic code. It was established permanently in the DNA/protein world with the establishment of the central dogma. Biological memory enables digital information to be stored, retrieved, and processed when needed. Codon–amino acids mapping created a permanent ‘memory bank’, which is manifested in the universal genetic code. The prebiotic memory bank is analogous to the memory bank in the machine language translation system. 

The information for making a specific protein is encoded in a single mRNA gene. We provided a model of how pre-mRNA and mRNA were created and encoded by orchestrating pre-tRNA/pre-aaRS and tRNA/aaRS molecules, respectively, before the advent of DNA transcription. Computer simulations and visualization are emerging technologies in the progress of molecular biology. Visualization may provide an instant, transparent, and intuitive understanding of the complex dynamics of biochemical processes and the origin of the first gene. 

### 2.4. Comparison between the Computer Information System and the Prebiotic Information System

There is stunning parallelism between biological information systems and human-made computer systems; nature invented these systems four billion years ago in a more subtle, complex, and sophisticated way and is still operating with high fidelity in all life. The amount of organization and coordination in a single autonomous protocell in the peptide/RNA world far exceeds the amount of organization in a modern computer program [9]. Here we show this uncanny convergence of prebiotic information systems of AIS, HIS, and DIS with similar information systems in computers that may shed new light on the origin and complexity of the biological information systems. 

## 3. Hierarchical Origin of Life and Information Systems

**Figure 1:**
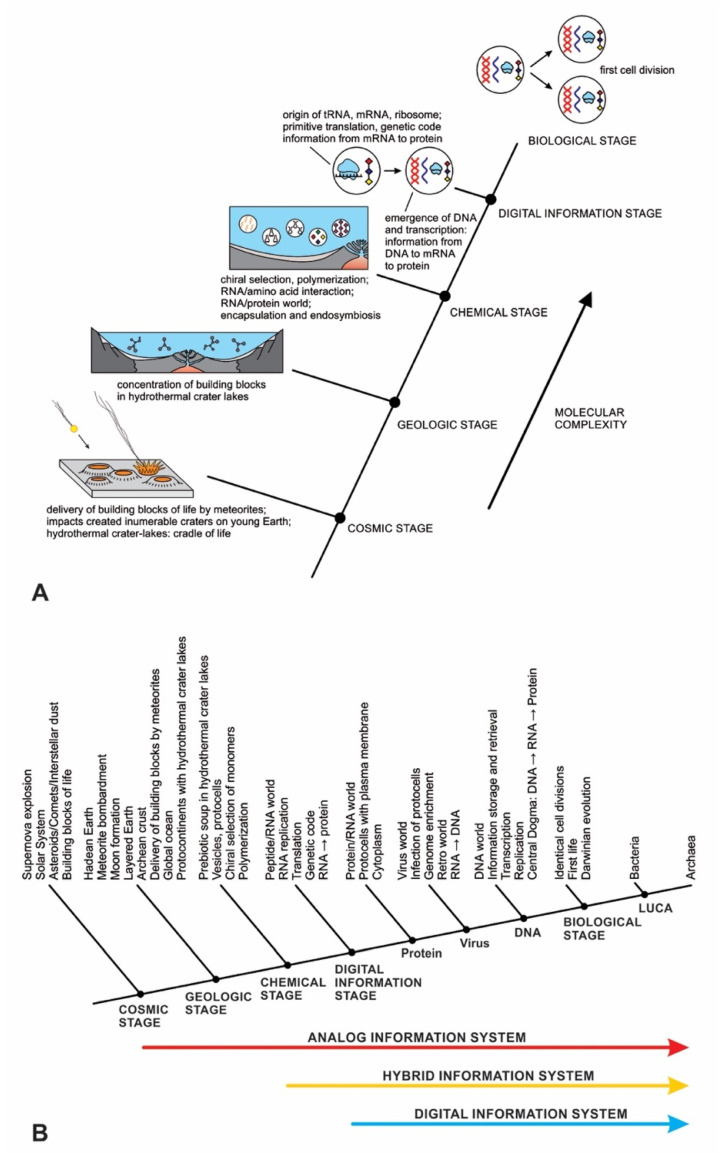
(**A**) the hierarchical origin of life, viewed as five ascending stages of increasing complexity, showing the biomolecules in the prebiotic world that led to the development of the first cells. These are the cosmic, geological, chemical, information, and biological stages—each higher-level acquired novel emergent properties. In the dark hot environments of hydrothermal crater lake basins, prebiotic synthesis led to first life. (**B**) the three ways of processing information in life are analog, hybrid, and digital, shown against the hierarchy of life.

In previous publications, we discussed the hierarchical origin of life in a bottom-up approach [12,13,14]. In our model, life arose about four billion years ago through five hierarchical stages of increasing molecular complexity in terrestrial hydrothermal crater basins, the likely cradle of life. These stages are cosmic, geologic, chemical, digital information, and biological. Figure 1 shows a schematic model for the origin of life and its information systems. An information-processing application to the origin of life is more robust than the purely chemical evolution of life. 

The origin of life is a unique product of two worlds: the building blocks of life from space and abiogenesis in the cradle of our planet. In the *cosmic* stage, a star explosion nearby the solar nebula cast the building blocks of life into interstellar space. During the Late Heavy Bombardment period, the comets and carbonaceous chondrites produced within that nebula transported water and organic molecules to young Earth. Carbonaceous chondrites, when impacted by young Earth, delivered a suite of building blocks of life and water that triggered abiogenesis. Meteorite impacts shipped these ‘seeds’ of life to young Earth along with water. Asteroid collisions created numerous hydrothermal crater lakes on the Archean crust, crafting cradles for prebiotic chemistry. 

## 4. Where Did Information Emerge in the Prebiotic World?

Information is the key property that distinguishes life from inanimate objects. However, what is information? Wiener succinctly suggested that *Information is information, neither matter nor energy* [15]. Additionally, its status as a physical entity remains obscure. Thus, what is information exactly? Information is intimately connected with matter and energy since both are required for its creation, initiation, and transmission. Life depends as much on the flow of information as on the flow of energy. Information flows like an invisible wave from the environment through biomolecules to create order out of chaos. Early cells could sense, compute, and make decisions: to increase in order to protect themselves against viruses and unfavorable environmental changes. Where did life’s information system begin? 

### Site for Life’s Information System

Information has a physical basis through thermodynamics. Life is an open system and interacts with its environment—life exchanges matter and energy with its environment and its information systems. Like energy, information can be transferred from one object to another in a living system. Living systems respond to the continuous environmental signals by complex computations they encounter. The information content of life can arise if the information in its environment falls. Shannon [16] formalized information concepts as a message, the transmission of the message, and the meaning of communication and information. He suggested that information allows the carrier of that information to make predictions with accuracy better than chance. The Shannon equation is formal for specifying a signal or message, the semantic aspect of communication and information. It approximates the physical space it may occupy. In this equation, the most random sequences give the highest possible entropy values (bits). When information is lost in a noisy entropy channel, the entropy rises. However, Shannon’s information entropy (H) is often confused with physical or thermodynamics entropy (S) because both concepts have similar mathematical forms but different meanings. Thermodynamics entropy characterizes a statistical ensemble of messages. Biological information, a form of thermodynamics entropy encoded in genomes, is fundamentally different from Shannon’s entropy. There is another dimensional aspect in biology: information has both a probabilistic and a linguistic context over an observable data set. Information in a biological context must exist within meaning [17]. Life is an open system and avoids decay by importing information or negative entropy from its surroundings. It grows by concentrating within itself and exporting entropy. The source of biological information is its environment [18].

The discovery in the 1960s and 1970s of wide assortments of biomolecules including hydrocarbons, sugar, amino acids, carboxylic acids, phosphorous, and four nucleobases—in carbon-rich asteroids, comets, and interplanetary dust particles supports the thesis of the extraterrestrial delivery of the building blocks of life rather than their endogenous production on the primitive Earth itself [19,20]. A study of the Murchison meteorite suggests these organic molecules developed analog information in the interstellar medium [21]. These building blocks of life were delivered to Earth by meteorites with an embedded analog information system (AIS). The origin of life may have had interstellar beginnings during planetary formations, but meteorite impacts jump-started life by transporting these crucial biomolecules to the Earth’s surface. The interstellar dust and meteorites provided the building blocks of life, but our Goldilocks planet (with a habitable zone from the Sun with the right temperatures for water to remain liquid) provided the ideal cradle for the biosynthesis that converted sterile molecules into living entities. There was a transfer of information site from the interstellar medium to the terrestrial hydrothermal crater vent on young Earth during the beginning of abiogenesis. 

In the cosmic stage, meteorites delivered building blocks of life and water to the young Earth during the tail end of the heavy bombardment [12,13,14]. A heavy bombardment of asteroids about four billion years ago created the crust of young Earth with innumerable craters, resembling the surface of Mercury and the Earth’s Moon. These high-energy meteorite impacts produced volcanically driven geothermal vents. These hydrothermal vents were filled with rainwater and the cosmic building blocks of life. 

**Figure 2:**
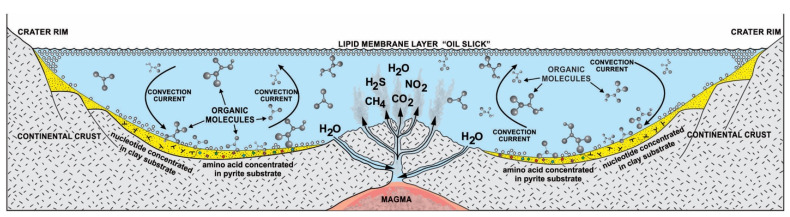
Cradle of life and its information system. Hydrothermal crater lakes in the Early Archean offered a protective haven for prebiotic synthesis. The boiling water was rich with building blocks of life. On the surface crater basin, lipid vesicles and hydrocarbons were buoyant like tars. The mineral substrates on the floor of the basin acted as catalytic surfaces for the concentration and polymerization of monomers. Convection currents thoroughly mixed the bubbling biotic soup. Some lipid vesicles by convective current went down to the crater floor and stuck to the mineral substrate, encapsulating biopolymers such as RNA and peptides. Hydrothermal vents provide heat, gases, and chemical energy, including thioester and ATP molecules.

Without some mechanisms to greatly concentrate cosmic biomolecules, more complex molecules could not be synthesized. Life probably began in freshwater hydrothermal crater lakes on protocontinents [12,13,14]. These interconnected crater lake networks of different sizes went through cycles of hydration during the rainy season and dehydration during the summer. Other than sunlight, heat from hydrothermal vents accelerated the dehydration cycle, especially in small craters. In order to begin prebiotic synthesis, the cosmic organic compounds were deposited and concentrated by convection current in the hydrothermal crater lakes. The convective current churned the prebiotic soup inside crater basins rich in organic compounds and caused simple chemicals to become more complex molecules. In this prebiotic soup, various organic compounds such as lipid molecules and various monomers such as amino acids, carbohydrates, and nucleobases were available to support the origin of life. Hydrothermal crater lakes encompass a multiplicity of physical, chemical, and mineralogical gradients conducive to prebiotic synthesis. Many of the chemical entities such as CO_2_, CH_4_, NH_2_, NO_2,_ and H_2_S that emerge from the hydrothermal vent of the crater lake are not in chemical equilibrium after rising water in which they are dissolved, cooled, and mixed with fresh water. As a result, these chemical entities enter chemical reactions with the cosmic organic molecules. Various energy sources such as heat, light, thioester, and ATP were available to start reactions in the vent environment from equilibrium [4]. This means that the mixture would not be stable, but instead, there would be a constant input of fresh organic compounds that were being constantly transformed by energy into other compounds. In this chaotic, far from equilibrium vent environment, life’s information system began to emerge along with prebiotic synthesis (Figure 2).

## 5. Analog World

The first vital step in abiogenesis was the synthesis and accumulation of abundant, carbon-based molecules as well as water. Life as we know it is based on six elements: carbon (C), hydrogen (H), oxygen (O), nitrogen (N), phosphorous (P), and sulfur (S)—typically abbreviated as CHONPS. These six elements are the building blocks of life: their covalent combinations make up most of the biological molecules on Earth.

We assert that life and its information systems coevolved in the hot, dark, and isolated environment of the hydrothermal crater basins that served as incubators four billion years ago. Cosmic molecules, big and small, acquired analog information during synthesis in space and performed specific functions. Primitive Earth favored an analog format to begin prebiotic synthesis from cosmic ingredients. Each cosmic molecule had its analog information system embedded as the study of the Murchison meteorites revealed the self-assembly of lipid vesicles [21]. As these molecules were concentrated in hydrothermal vents, they increased molecular complexity enriching their information content. The analog information systems in cosmic molecules became elaborated, modified, and fine-tuned during abiogenesis in the crater vent environment. In an analog system, information is manifested in a continuous variable composition of the molecular milieu in a prebiotic soup.

Many critical biomolecules were delivered by meteorites during the early bombardment and were available in the hydrothermal vent environment. These biomolecules are lipids (CH_3_(CH_2_)_14_COOH, etc.), sugars (C_6_H_12_O_6_, etc.), amino acids (C_3_H_7_O_2_N, etc.), and nitrogenous nucleobases (such as adenine, guanine, cytosine, and uracil). These four classes make up the three essential parts of the living cell: the cell membrane, proteins, and nucleic acids. Out of these three essential biomolecules, cell membranes and proteins contain analog information, and nucleic acids are the carrier of digital information.

The appeal to the analog framework in prebiotic energy sources is that it is easier to synthesize in the hydrothermal vent environments under abiotic conditions. The origin of life depicts how a living system can emerge from a chaotic assemblage of building blocks of life in the hydrothermal vent environment. It would have required the organization and selection of just the right combinations of the smaller molecules into larger macromolecules. Many molecules were discarded from the prebiotic synthesis, and few were selected. We started by analyzing the simpler chemical components of emerging life, those cosmic biomolecules that might have been selected, concentrated, and organized into the essential structures of life.

We identified eight major steps in chemical evolution along with its analog information system during prebiotic synthesis in a hydrothermal crater vent environment. These are (1) chiral section of monomers, (2) conversion of nucleobases to nucleotides, (3) self-assembly of lipid chain into bilayer membrane, (4) polymerization of monomers on mineral surfaces, (5) encapsulation of polymers, (6) insertion of peptides into lipid bilayer membrane, (7) protometabolism, and (8) growth and division of protocells.

### 5.1. Chiral Selection of Monomers

Monomers such as amino acids and carbohydrates that are essential to life were first recruited from cosmic building blocks to begin prebiotic synthesis in hydrothermal crater basins. Amino acids are polymerized into long protein chains, and simple carbohydrates such as ribose sugar link up with phosphate groups to form RNA. These molecules came from space in a racemic mixture of chiral molecules (equal amounts of left-handed and right-handed enantiomers). Many molecules come in a racemic mixture or mirror-image forms, known as right-handed and left-handed. The two forms of a chiral molecule, or enantiomers, have identical chemical and physical properties, but the way each interacts with other chiral molecules may be different. Both forms of a given molecule are created in a chemical process, but a biological process selects just one homochiral molecule. Life is based on patterns of homochirality, such as left-handed amino acid (L-amino acid) and right-handed ribose sugar (R-ribose). This asymmetry, L-amino acids and R-ribose, is a unique signature of life on earth, but its origin in prebiotic synthesis remains unanswered. The process by which life became homochiral appears early in the prebiotic synthesis in the vent environment.

**Figure 3:**
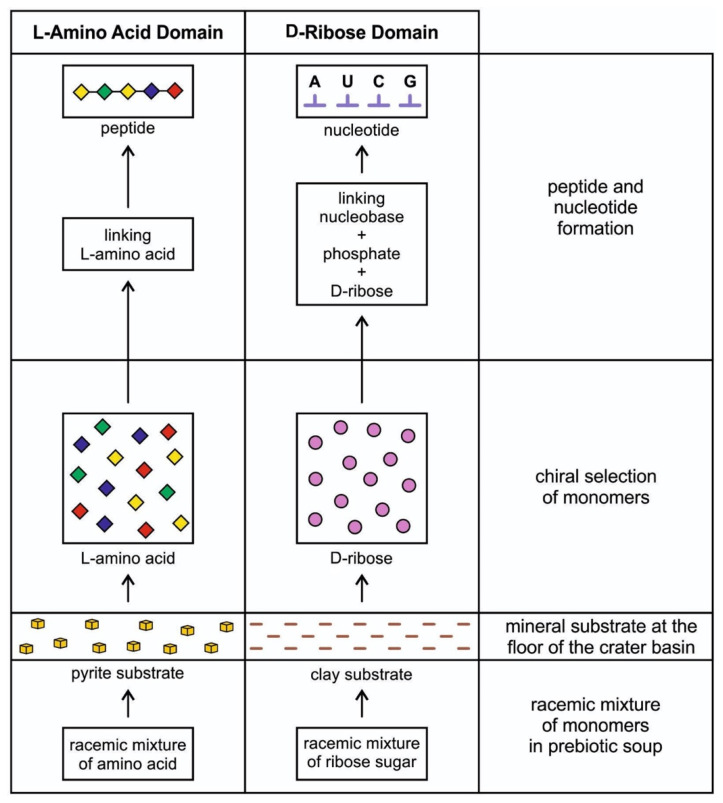
Chiral selection of monomers such as L-amino acids and D-ribose sugar from the racemic mixture on the mineral substrate floor of the hydrothermal crater vent environment. A short chain of the peptide can be formed by linking a few L-amino acids to each other via peptide bonds by condensation reaction. L-amino acids become monomers of proteins. On the other hand, D-ribose joins with a phosphate molecule to form the backbone of a nucleobase; these three molecules join to form a nucleotide, the monomer of RNA.

The idea that life is chiral had puzzled scientists for more than 170 years when Louis Pasteur discovered chirality. Although some amino acids within meteorites were suggested to have an enantiomeric excess of L-amino acids, chiral selectivity most likely occurred and amplified in a local chiral environment of a hydrothermal crater vent during prebiotic synthesis. At the crater floor basin, the mineral surface of clay and pyrite formed—the clay from impact melt and the pyrite from a hydrothermal vent [14]. Another source of clay was the impactor itself, carbonaceous chondrite. Clays such as smectites drove protometabolism and had the catalytic ability. Amino acids and nucleotides are adsorbed on the clay surface at the floor of the crater basin and subsequently polymerize [22]. The granitic terrain of the crater basin provided various minerals (Figure 2). It is possible that the crystal faces of enantiomorphic pairs of crystals, such as quartz, feldspar, diopside, and calcite, separated chiral molecules from racemic mixtures of prebiotic soup [23]. Experiments showed that the chiral faces of these crystals attract chiral molecules of similar handedness. For example, the left-handed faces of calcite may have concentrated left-handed amino acids and vice versa. Perhaps the chiral crystals of the vent environment’s mineral substrate facilitated the asymmetry of chiral biomolecules. These chiral molecules were adsorbed and concentrated on the mineral surfaces at the crater basin. The molecular preference of AIS played a dominant role in the chiral selection of monomers. The chiral selection was a critical step for prebiotic synthesis (Figure 3).

Protein is a fundamental subsystem of life, and it plays a vital role in life’s functioning. Every single one of these molecules—from amino acids to peptides to enzymes—is made of L-amino acids. Similarly, carbohydrate is another crucial component of life. Each of these molecules—from glucose to ribose sugar in RNA to deoxyribose sugar in DNA—is a specific right-handed chiral form. The selection of D-ribose and L-amino acid in life synthesis remains unknown. Why is the L/D symmetry broken by life? Is it a sheer accident in the environmental condition during abiogenesis? Or is chiral purity required for biological function? Only the L/D protein/carbohydrate combination is present in life. Perhaps D-sugars preferred L-amino acids by molecular attraction. We do not know the answer. De Duve [4] suggested that the initial choice of R-ribose for the synthesis of nucleotides dictated the choice of L-amino acid for the assembly of peptides. He reduced the choice to the solitary ribose molecules instead of nineteen amino acids (except for glycine, where the side chain is H). This molecular choice or attraction might be an example of pure chance or a ‘frozen accident’. A chance occurrence of prebiotic chemistry resulting from an asymmetric, local physical environment triggered an initial chiral selection.

Experimental evidence corroborates the speculation of De Duve that R-ribose might have selected the L-amino acid from the racemic mixture that is incorporated into proteins [4]. In protein synthesis, aminoacyl-tRNA synthetase (aaRS) attaches a correct L-amino acid to a specific tRNA molecule to form an aminoacyl-tRNA, which ensures the use of L-amino acid in protein synthesis. Tamura and Schimmel demonstrate that the non-enzymatic aminoacylation reaction of an RNA minihelix has a chiral preference for L-amino acid over D-amino acid [24]. The rationale for using minihelix in experiments is that it may be a precursor to tRNA and might represent a transitional stage of aminoacylation. Chemical geometry in RNA minihelix might be the underlying mechanism for chiral selection of L-amino acid [25].

Asymmetric autocatalysis can drive spontaneous symmetry breaking between L and D enantiomers. The most likely form of autocatalysis in biomolecules is templating of oligonucleotides, as it was shown that homochiral oligomers are good templates and oligomers of mixed chirality are not. This leads to chiral symmetry breaking when the templated ligation is high [26].

Chirality is an essential component of biochemistry for molecular recognition and replication processes and would seem to be essential for abiogenesis. We speculate that the molecular preference of D-ribose for L-amino acid is linked to one another by stereochemistry and is an early manifestation of analog information. The transmission and amplification of handedness and its embedded information from the molecular to supramolecular level are required for abiogenesis. The complementary nature of these two classes of molecules was required for creating informational biomolecules: D-ribose for nucleotides and RNA and L-amino acids for peptides and proteins. Both environmental and chemical factors played essential roles in the emergence of molecular homochirality.

### 5.2. Conversion of Nucleobases to Nucleotides

Although nucleobases such as adenine, guanine, cytosine, and uracil might have come from space [19,20] and deposited and concentrated in the hydrothermal vent, nucleic acids such as RNA use nucleotides for polymerization. Prebiotic nucleotide synthesis from the assembly of a nucleobase, a right-handed ribose, and phosphate is crucial to understanding the origin of life and its information systems. Becker et al. [27] demonstrated a plausible prebiotic process for the concurrent synthesis of purine and pyrimidine ribonucleosides and ribonucleotides, driven solely by wet–dry cycles. In the crater lake environment, a wet/dry cycle was provided by the exposed sloping rim, where each nucleobase was linked to sugar to form nucleoside, which in turn was linked to a phosphate group to form a nucleotide. A nucleotide with a sugar-phosphate backbone became a monomer for polymerization to RNA-like molecules. The wet/dry cycle of AIS was instrumental in forming nucleotides that would lead to the origin of RNA by polymerization.

### 5.3. Self-Assembly of Lipid Chain into Bilayer Membrane

**Figure 4:**
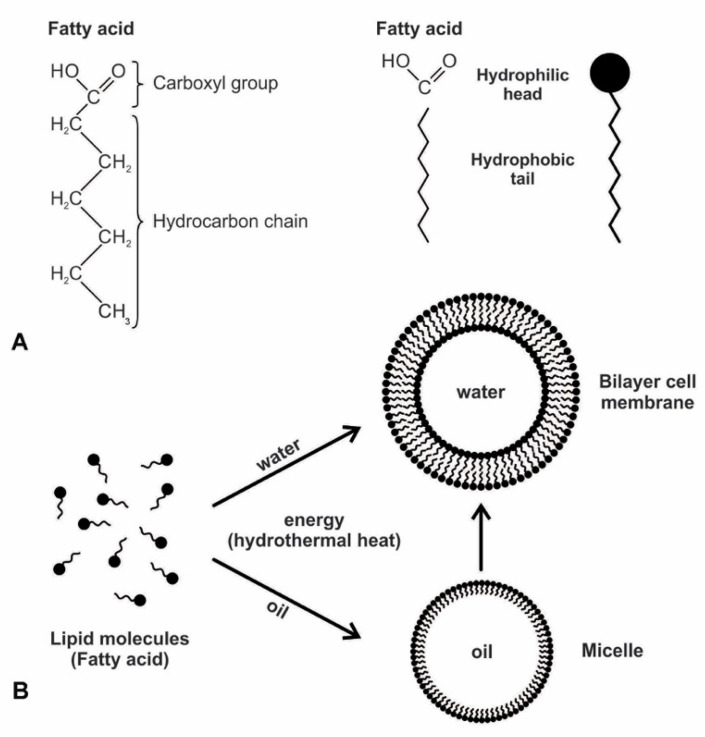
Amphiphilic compounds like fatty acid can self-assemble into cell-sized vesicles bounded by a membrane. (**A**) the polar simple fatty acid was likely a major component of the early prebiotic cell membrane due to its ability to form a vesicle. It has a hydrophilic head and a hydrophobic tail. (**B**) as a monolayer, a micelle can only trap oils, not water, and thus cannot be a precursor to the cell. A bilayer vesicle that trapped water and water-soluble molecules must have given rise to the cell membrane.

The next stage of the analog information system is the spontaneous formation of the amphiphilic bilayer membrane. Every living cell is surrounded by a double layer of the plasma membrane that separates the cell’s interior from its outside environment. It is selectively permeable, permitting certain molecules to enter and exit the cell. During the early stage of abiogenesis, the primitive membrane was not sophisticated as a plasma membrane but was constructed by simple lipid molecules such as fatty acids. These lipid molecules were delivered by carbonaceous chondrites and were accumulated in the hydrothermal vent environment, floating at the surface, or attached to the substrate of the crater floor. These lipid molecules were amphiphilic, in which the hydrocarbon chain loves oil, and the carboxyl group loves water. They could self-assemble into bilayers in cell-sized membranous vesicles that spontaneously formed essential boundary structures of protocells (Figure 4). The spontaneous reaction produces order from the disorder with a reduction in entropy. The subunits interact due to molecular forces such as covalent bonds, hydrogen bonds, hydrophobic effects, electrostatic interactions, and Van der Waals forces. When molecules are concentrated above a certain threshold, molecular forces can drive the self-assembly of fatty acids into membranous compartments bounded by lipid bilayers if the chain lengths are ten or more carbons long [21]. Self-assembly proceeds spontaneously and releases energy (an exergonic process) and does not require anything other than the availability of the component molecules themselves.

The secret of membrane construction is the lipid bilayer. The unusual interaction of lipids with water makes them very valuable. They are waterproof and energy-rich. While lipids can spontaneously form a monolayer or a bilayer, due to the polar nature of amphiphiles, only a bilayer could have served as the protocell of the membrane. The hydrophilic head is pulled to the water molecules, while the hydrophobic tails are attracted to each other because they are repelled by water. The bilayer membranes are stabilized by this hydrophobic effect and the Van der Waals interactions between tails [28,29]. A closed monolayer creates a micelle whose external surface is always composed of the hydrophilic heads; the internal surface, consisting of hydrophobic tails, renders a monolayer unable to contain water. A bilayer avoids this by having both exterior and interior surfaces that are hydrophilic. Such a vesicle can trap moisture and water-soluble molecules in the cytoplasm, such as peptides, ribozymes, RNAs, sugars, and proteins. Life must have arisen from this bilayer of the biomolecule. Membranes consisting of fatty acids are reasonably permeable to smaller polar nutrients such as nucleotides and even to charged species such as ions [21]. Prebiotic vesicles were undoubtedly composed of complex mixtures of amphiphiles. Compared to membranes composed only of single molecular species, such as fatty acids, those of mixed amphiphiles often have superior stability and tolerance over a wide range of pH and ionic conditions.

### 5.4. Polymerization of Monomers on Mineral Surfaces

The next stage of the analog information system is the non-enzymatic polymerization of monomers such as L-amino acids and nucleotides into protein and RNA-like molecules by condensation reaction. Peptide and ester bonds link these polymers, respectively. The polymerization reaction in both cases is thermodynamically uphill, favoring hydrolysis. These monomers are water-soluble and need mineral substrate or a wet and dry cycle to polymerize. The production of their corresponding polymers requires two distinct steps: the correct molecules first must be concentrated and then assembled into the desired structure. The chemical bonds that link monomers into polymers are formed by a reaction called condensation, in which a water molecule is removed from being between chemical groups of monomers. Condensation reactions synthesize random assortments of polymers. Complex and highly organized molecules are not expected to form spontaneously from simpler constituents; monomers must absorb energy to link together. In the energy-rich hydrothermal vent environment, available free energy could have driven polymerization through a condensation reaction. The mineral surface of the crater basin provides a viable catalytic mechanism for polymerization in a hydrothermal setting. Molecular adhesive forces between the microscopic layers of minerals first brought disparate monomers close together, allowing them to link into polymers. Recent experiments suggest that monomers incubated by tiny mineral particles such as clay or pyrite could have polymerized these monomers, analogous to solid-phase synthesis [21,22]. These minerals could thus have provided the scaffolding upon which monomers formed polymers such as RNA and polypeptides.

**Figure 5:**
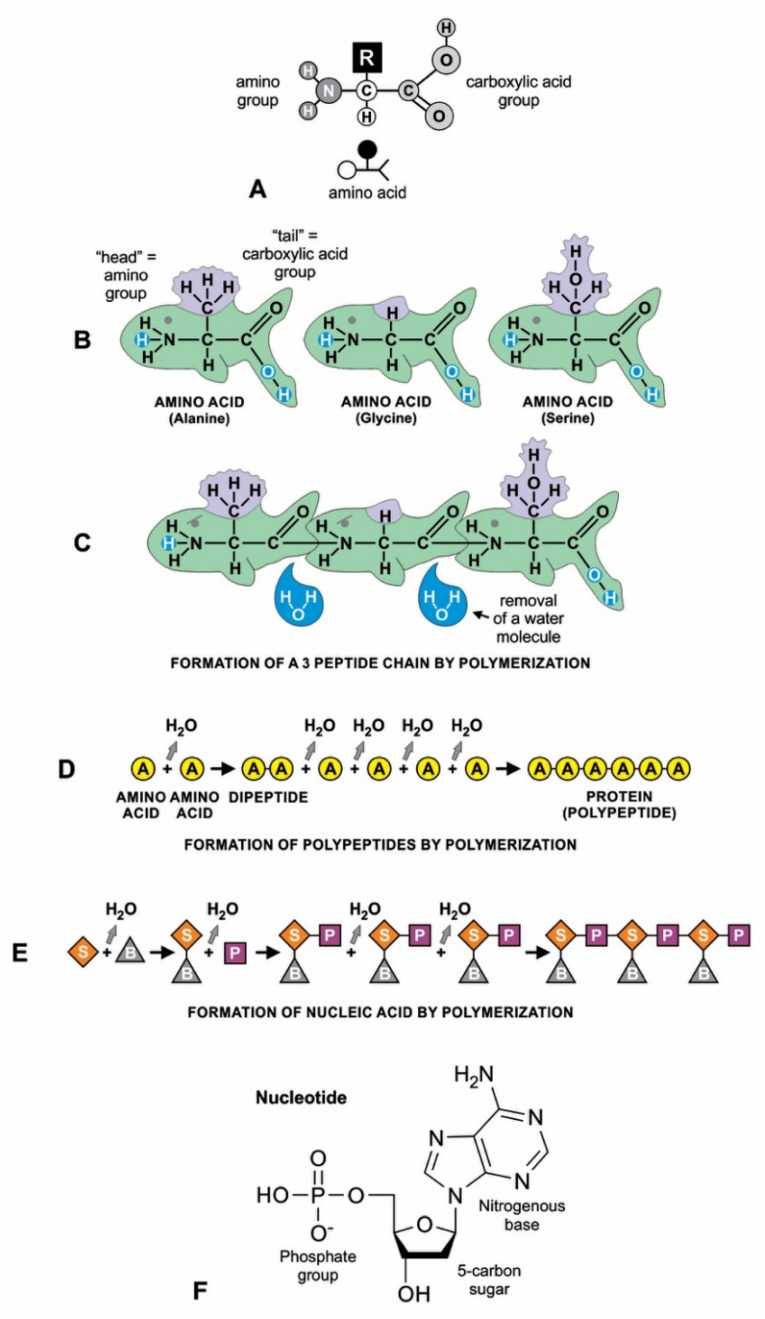
Condensation reaction on mineral surfaces, where activated monomers drive endergonic polymerization reactions; (**A**) amino acid structure; all amino acids have the same general configuration: a central carbon bonded to an amino acid functional group, a carboxyl functional group, a hydrogen atom, and a side chain, or R-group. (**B**–**D**) How amino acids polymerize to form polypeptides by peptide bonds; (**B**) the resemblance of an amino acid to a fish helps differentiate its parts. The three amino acids chosen as examples are incredibly similar: each possesses a carboxylic acid group (the ‘tail’) and an amino group (the ‘head’). However, they differ in the ‘dorsal fin’ (R-group of amino acid), which determines the kind of amino acid (here, alanine, glycine, and serine). (**C**) three molecules of amino acids can polymerize into a polypeptide by linking the amino group of one with the carboxylic acid group of another. This reaction forms a water molecule through the combination of a hydrogen ion (H^+^) discarded from the carboxyl group and a hydroxyl group (OH^_^) discarded from the amino group). (**D**) shows how a longer chain of amino acids (i.e., a polypeptide) can be formed by removing a water molecule from each link; mRNA-directed protein molecule is also formed similarly by linking amino acids in ribosome during translation. (**E**) nucleotides can join into an RNA molecule by linking the sugar (S) and phosphate (P) molecules with the backbone of the ribonucleotide bases (B). (**E**) the linking of nucleotides into RNA was accomplished by dehydration; (**F**) shows the three components of a nucleotide (phosphate group, sugar, and nitrogenous base) in more detail.

Amino acids have a simple molecular structure consisting of a carboxyl group, an amino group, and a variable R group attached to a carbon atom (Figure 5A). They form a peptide or protein chain when a covalent bond forms between the carboxyl (C) group of one amino acid and the amino (N) group of another, which removes water (Figure 5B,D). The C–N bond that results from this condensation reaction is called a peptide bond. Both peptides and proteins are composed of L-amino acids, but they differ mainly in size: a peptide (a string of two or more amino acids) is usually much shorter than a protein (at least 50 amino acids in a variety of configurations). The random sequences of amino acids in proteins formed by condensation reaction were not enzymes and would have little use in abiogenesis; they would decompose into amino acids by hydrolysis. On the other hand, small peptides would form a partnership with RNA molecules [21].

Clay minerals have a large adsorption capacity to concentrate and catalyze organic molecules for the polymerization of nucleotides because of the ordered arrangement of clay mineral particles, as well as its unusual charge properties [22]. RNA can be assembled end-to-end into linear molecules of nucleotides on the clay mineral surface, just like amino acids when polymerized into polypeptides (Figure 5E). The polymerization reaction involves forming a bond between the phosphate group of one nucleotide and the hydroxyl group of the deoxyribose sugar component, resulting in a phosphodiester bond. Similar to the peptide bond that joins amino acids, this bond results from a condensation reaction and thus removes a water molecule from the bonded nucleotides.

Here we distinguish two kinds of polymerization of RNA molecules: non-enzymatic (or abiotic) and enzymatic (or biotic). Before the emergence of protein enzymes, all RNA molecules had to be synthesized abiotically. These abiotic RNAs would have been noncoding RNAs, meaning they lacked the genetic triplet code of biotic RNA and could not encode proteins. They represent *quasispecies* out of which many species of RNA (perhaps ribozymes, tRNA-like molecules, and other ncRNAs) may have developed.

### 5.5. Encapsulation of Polymers

The next stage of analog information is the encapsulation of polymers for prebiotic synthesis. The encapsulation of RNAs and polypeptides along with amino acids and nucleotides must have occurred very early in the development of life in the chemical evolution, soon after the availability of these polymers in the hydrothermal vent environment. Encapsulation first requires the bilayer membrane to open, allowing larger molecules to enter. RNAs and peptides cannot permeate lipid membranes. How then were they encapsulated within the lipid bilayer? Two different processes were suggested for the encapsulation of polymers during terrestrial prebiotic synthesis. In the first model, continuous wet/dry cycles in temporary hydrothermal ponds allow condensation on the surface of the lake. The hydration/dehydration cycle permits the formation of vesicles with encapsulated polymers in a hydrothermal pool rich in fatty acids [29]. Upon the completion of the dehydration cycle, amphiphiles could self-assemble into dried multilamellar structures containing monomers between their layers. At the same time, condensation reactions polymerize amino acids and nucleotides into peptides and RNAs, respectively, all while preserving the lipid bilayer. In the following hydration cycle, vesicles are formed when the dry multilamellar matrix interacts with the water. Some vesicles would come empty, and others would contain polymers.

**Figure 6:**
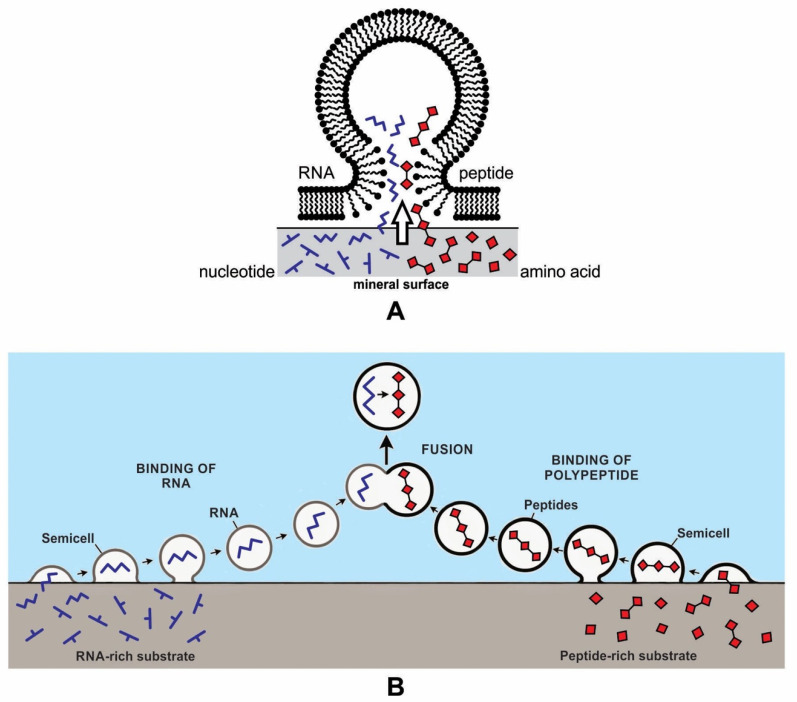
Two possible models of the encapsulation of polymers by simple fatty acid membranes on the mineral surface. In model (**A**), both RNAs and polypeptides are brought together in the same vesicle. In model (**B**), RNA and peptides are encapsulated separately on the crater basin, then fused in the aqueous environment.

We previously suggested an alternative model for the encapsulation of RNAs and polypeptides on the mineral surface of the hydrothermal vent environment [12,13]. Mineral surfaces would have been able to concentrate and polymerize monomers and thus produce RNA and peptides (Figure 6). The hollow lipid membranes would stick to the mineral substrate like tiny blisters, providing access to a wide range of polymers as well as other biomolecules. In this model, the vesicle crowded at the crater floor on the mineral surface could trap RNA and polypeptide from the adsorbed surface of the clay and encapsulate them, bringing these two components together to generate a protocell-like structure. As these protocells were released from the mineral surface, their polymers became encapsulated, ready to participate in further chemical reactions. This initial cooperation between the encapsulated polymers might even be called the origin of life. Within these newly formed protocells, genetic material could reside and be replicated, and metabolism could occur. From there, protocells could begin to develop other biotic functions, such as the self-assembly of boundary membranes, transport of monomers, and encapsulation of polymer systems capable of growth and of developing an information system [30]. Encapsulation of polymers by a lipid bilayer membrane led to the development of protocells.

During encapsulation, the vesicles would capture polymers like RNA and peptides and prebiotic soup containing these molecules to maintain the vent environment. This is the beginning of primitive cytoplasm, an aqueous medium, inside the protocell. This primitive cytoplasm became the reservoir of various polymers and other chemicals when needed (Figure 6). Encapsulated systems of molecules would be essential for life for abiogenesis in a protected environment allowing natural experiments to occur. This is the beginning of the age of the protocell. However, such a lipid membrane should have a crucial weakness in that protocell surrounded by the lipid membrane cannot survive for a long time because it would be difficult to incorporate enough hydrophilic organic compounds through the lipid membrane. This deficiency of lipid membrane was improved with the development of peptide channels that allowed hydrophilic compounds and other nourishment from the environment to enter the protocells. If the protocells incorporated RNA molecules, they could undergo a primitive form of growth and division [28].

### 5.6. Insertion of Peptides into Lipid Bilayer Membrane

**Figure 7:**
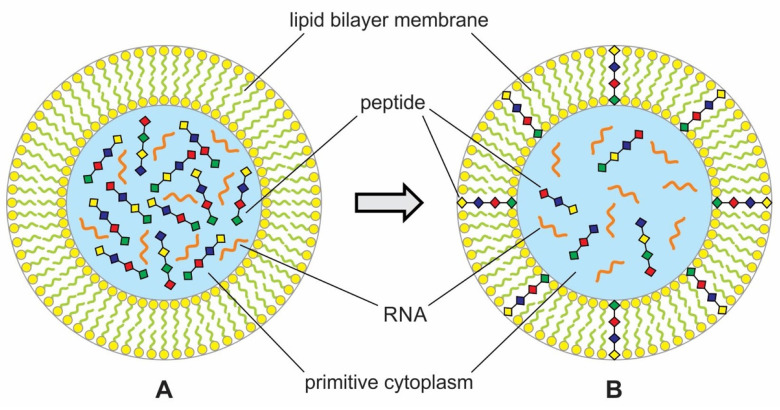
Primitive protocell enclosing assemblages of peptide and RNA molecules. (**A**) Encapsulated polymers such as peptides and RNA and prebiotic soup to create primitive cytoplasm. (**B**) Some peptides were inserted into the lipid bilayer to enhance permeability in the protocell. The peptides would produce ion-conducting channels through the bilayers that allow phosphate, thioester, ATP, and other nutrients such as amino acids to enter the cell. Molecular crowding inside primitive cytoplasm would encourage symbiotic relations between peptides and RNAs.

Lipid bilayers were a barrier to diffusion for water-soluble solutes such as amino acids and phosphate or simple ions like sodium (Na^+^) and sodium (K^+^). The bilayer barrier is essential to maintain the integrity of polymers, but nutrients from the environment by diffusion were also necessary for the growth of protocells. Hladky and Hayden [31] suggested a mechanism by which the permeability of protocells can be enhanced by inserting peptides such as antibiotics called gramicidin. These pore-forming peptides can spontaneously insert across a lipid bilayer. Some peptides could be inserted into the lipid bilayers in prebiotic synthesis to create a channel for ions and soluble solutes (Figure 6). Deamer [21] elaborated this concept of peptide insertion into lipid bilayer to enhance permeability in protocells. This insertion of peptides into lipid bilayers may be a precursor to the plasma membranes, where proteins are inserted into phospholipid bilayers to transport a given solute across the bilayer barrier. At this stage, a rudimentary form of signal transduction had evolved from the environment to the cytoplasm via the peptide channel (Figure 7).

### 5.7. Protometabolism

Prebiotic chemical reactions in vent environments were the forerunner of the present-day metabolism, called protometabolism [4]. Metabolism follows metabolic pathways, the flow of chemical reactions, each catalyzed by a series of enzymes and acting on the previous enzyme’s product. Since enzymes were not available before the synthesis of proteins, minerals containing metals such as iron sulfide probably functioned as catalysts for protocells. The water of the vent environment was rich in ferrous iron and transition-state metals, such as ions of magnesium, copper, and zinc, compounding the catalytic capabilities of the iron-rich clays of the crater. Mineral catalysts may have played important roles in establishing early metabolism. Apatite might have helped in the building of the cell membrane because of its phosphate content. Metal ions of Fe, Mn, Zn, and Cu also were available in the vent environment, which helped mediate catalysis. At the crater floor, crystalline surfaces of common rock-forming minerals, such as pyrite and montmorillonite, enhanced protometabolism by polymerizing nucleotides into RNAs and amino acids into peptides [4,21,22]. The metabolic activity of these early peptides became improved with the availability of phosphates.

De Duve suggested a high-energy thioester-based protometabolism, which follows pathways not dissimilar to modern metabolism, in which thioesters also play a crucial role [4]. Thioesters are energy-rich, highly reactive compounds that, due to their ATP-like ability to store chemical energy and release it when thioester bonds are hydrolyzed or phosphorolyzed, can be used as an energy currency themselves. Throughout the living world, energy circulates almost entirely in the form of a single chemical entity, known as ATP (adenosine triphosphate). Hydrothermal vents produced a continuous stream of various chemicals and energy, such as ATP, facilitating the chemical and catalytic reactions of cosmic ingredients [32]. ATP played an important role in primitive metabolism. A sophisticated protometabolic AIS was developed to make use of energy, ions, signals, and nutrients via the peptide channel.

### 5.8. Growth and Division of Protocells

Membrane-bound protocells containing a set of monomers and polymers could grow and divide. Such protocells could acquire resources and energy from the environment. Under laboratory conditions, membrane vesicles composed of fatty acids can grow and divide and hint at a solution to the primitive protocell division mechanism. Lipid vesicles extracted from the Murchison meteorite undergo spontaneous primitive cell division in the laboratory, with no external forces acting upon them [21]. When a mixture of these cosmic vesicles, amino acids, and nucleic acids was shaken, the vesicles trapped the organic molecules inside them and began to interact. This suggests that vesicles can take substances from outside themselves through their lipid walls to build new walls and new contents. A large vesicle mimics a primitive kind of cell division. With the development of the peptide channel, the protocells could obtain nutrients and lipid components from the environment and could grow and divide.

**Figure 8:**
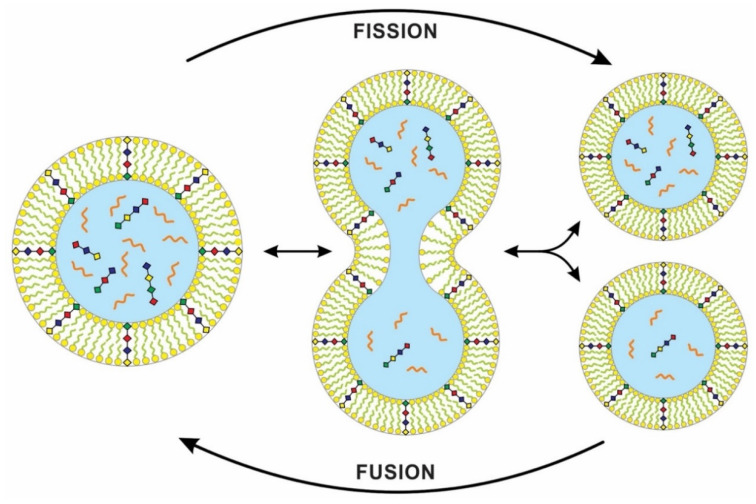
Fusion and fission of lipid bilayers with inserted peptide molecule (see Figure 7 for explanation). The peptide channels allowed nutrients, lipid components, and energy from the environment to enter protocells by diffusion for growth and division. These protocells form flexible, semi-permeable vesicles, capable of dividing into two such daughter vesicles or of joining with another without any moment of losing their structural continuity. Unlike living cells, the division of protocells is asymmetric, where daughter cells might inherit an unequal amount of cytoplasmic content. The transfer of information from parent to daughter cells is vertical. The cellular division of first cells inherited this property of protocells, but DNA replication created identical daughter cells.

The physics of ‘chemically active’ droplets, which cycle chemicals in and out of the surrounding fluid, may shed light on the origin of protocell division [33]. The team studied a theoretical model for the behavior of a liquid droplet in a chemically disequilibrated system. This ‘active droplet’ behavior differs from the passive and more familiar tendencies of oil droplets, which join into bigger droplets without dividing. On the other hand, these chemically active droplets can grow to a stable size by taking resources from the environment. Droplet growth eventually leads to instabilities linked to the changing shapes of the droplets. The droplet keeps elongating and pinches in the middle, which has low surface areas. Eventually, surface tension causes it to be split into a pair of droplets (Figure 8). This process of dividing droplets somewhat mimics the spontaneous vesicle division from the Murchison meteorite [21].

In a laboratory simulation, a genome-rich vesicle increased in size at the expense of an empty vesicle. When its greater size imposed too much osmotic stress, pearling instability developed, and the stretched vesicle divided into two, each daughter vesicle retaining some of the original genomic contents [34]. Recent work on model protocell membranes demonstrated that vesicles could grow as filamentous structures and divide spontaneously under mild shear forces. With photochemical stimulation, a robust ‘pearling’ mechanism produces many small daughter vesicles [35]. Self-replicating membranes can divide spontaneously or under the influence of external environmental forces [36], and high environmental shear forces can cause vesicles to divide. In a similar way, a protocell with cytoplasm can grow and divide into two daughter cells with identical cell membranes. However, the cytoplasmic division in the daughter cells that lacked a digital genetic system may be unequal. These differences can be viewed as analog equivalents to mutations [3].

Synthetic biologists use simple ‘protocells’ to study the origin of cell division, but previous models could not reproduce both the genome and the membrane sustainably. Kurihara et al. [37] proposed a recursive self-proliferating model protocell that represents a step towards the eventual production of model protocells that can mimic cell division. They used a novel system, fusing the self-reproducing vesicles with feeder vesicles, thus allowing the vesicle composition to be sustained over multiple generations. Because of competition, the larger vesicle grows more quickly and fuses with the feeder vesicles. Therefore, feeding the protocells by vesicle fusion offers a practical pathway for indefinite self-reproduction (Figure 8).

### 5.9. Summary of the Analog Information System

**Figure 9:**
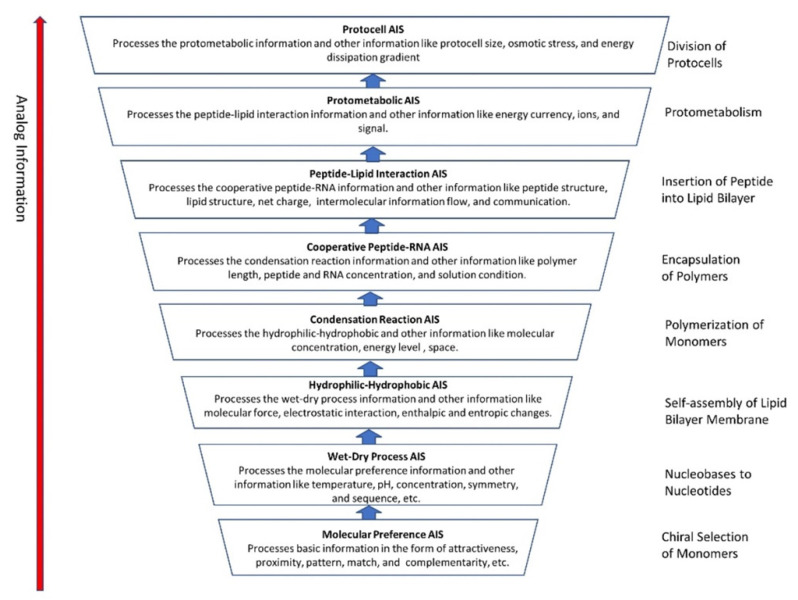
Hierarchical evolution of the Analog Information System (AIS) in the early stage of peptide/RNA world. The most basic AIS is termed ‘Molecular Preference AIS’. The higher-level stage AIS is built upon the lower-level AIS. For example, the next stage of AIS, the wet–dry AIS, subsumes the molecular preference AIS, and so on.

Here we summarized the hierarchical evolution of the analog information system (AIS) in the peptide/RNA world (Figure 9). The molecular preference that AIS uses the most is the basic mechanism of sensing and processing information. These mechanisms include detection of molecular concentration and temperature, natural attraction, physical proximity, simple structure fit, lock-and-key, etc. A surface-mediated mechanism of ‘structural face match’ between the surface and chiral molecules was used by the molecular preference AIS to select monomers. The higher-level biological analog information systems are built cumulatively on the lower-level analog information systems.

### 5.10. Analog Information Systems Reached a Cul-De-Sac during Abiogenesis

Analog information systems accomplished a remarkable feat in early prebiotic synthesis but could not proceed any further without the help of digital information systems. The solitary journey of analog information systems would fail to generate first life. However, it had created the nucleotides with Watson–Crick base-pairing rules to perform critical tasks of making hybrid use of information. The hybrid use of information involves processing information in both analog and digital forms. In turn, a hybrid information system would lead to a digital information system that would require sequences and coding rules.

## 6. Hybrid Information System

The close symbiosis between peptides and nucleic acids in modern cells indicates a functional co-evolution between two polymers that led to the beginning of the peptide/RNA world. A molecular replicator with two components, peptide and RNA, played a critical role in building the hybrid information system with a high degree of specified complexity. A hybrid information system makes use of information in both forms—the analog form and digital form. It is analogous to a hybrid car powered by fuel in two forms—the gasoline form and the electricity form. The hybrid information system would enhance and perfect digital computing and give rise to digital information systems (DIS).

Coding RNAs generally refer to mRNA that encodes proteins—the latter act as various components, including enzymes, cell structures, and mixed-signal transductors. mRNAs play key roles in the Digital Information System (DIS). In contrast, noncoding RNAs (ncRNAs) dominate the Hybrid Information System (HIS). They can form abiotically in the vent environment by polymerization of nucleotides. The ncRNAs belong to several groups and accomplish a variety of biological functions. The role of noncoding RNAs has increased attention in abiogenesis. Noncoding RNAs are not translated into proteins, but they are involved in making translation machines. Transfer RNAs (tRNAs) form an adaptor molecule between mRNA and protein. As discussed in later sections, they would play critical roles in creating coding RNAs (mRNAs). The ribosome consists of more than 60% ribosomal RNA, which is made of three ncRNAs in bacteria.

The evolution of hybrid information systems during prebiotic synthesis must consist of reasonable elementary steps. Each step confers a distinct added advantage that leads to the efficient use of digital information. We highlighted the salient features of the hybrid information systems during the major stages of abiogenesis. These stages are (1) base-pairing and RNA replication; (2) the emergence of noncoding RNA molecules such as ribozymes and tRNAs; (3) ribozyme–amino acid interaction and the origin of a bridge peptide; and (4) the origin of ribosomes. We discussed these processes in detail in our previous paper [35]. Here we outlined the main features of these processes to highlight the evolution of hybrid information systems.

RNA is a versatile molecule with many variants and functions. It is free to take any kind of shape as a single chain. Because of its architectural flexibility, a single-stranded RNA molecule could give rise to different species of noncoding RNAs, such as ribozymes, transfer RNA (tRNA), and ribosomal RNA (rRNA). Each species developed a unique configuration, attribute, and supply of information, in response to the specific amino acids with which it interacted. The coordination of different kinds of noncoding RNA molecules signals the passage from analog information to hybrid information. Here we traced the origin and function of other species of noncoding RNAs in the peptide/RNA world. Many of these critical roles and functions of ncRNAs in the peptide/RNA world are thought to be molecular fossils, relics, or lost with the emergence of the first cells, and their current roles remain mainly in the regulation of information flow from DNA to protein.

### 6.1. Base-Pairing and RNA Replication

Non-enzymatic chemical synthesis of RNA offers a possible link between analog and hybrid information systems. The non-enzymatic RNA replication is a transitional stage between the prebiotic origin of nucleotides, which are the building blocks of RNA, and the synthesis of an RNA chain by RNA polymerase ribozymes that could catalyze its own replication. However, significant gaps remain in our knowledge about how RNA replication happened before the appearance of an RNA replicase, a ribozyme.

The first replicable molecules in abiogenesis consisted of RNA. The linear sequence of nucleotides in an RNA molecule usually occurs in a single strand made up of a sequence of the four bases—adenine (A), uracil (U), cytosine (C), and guanine (G). Unlike polypeptides, polynucleotides can directly replicate exact copies of their sequences by the complementary Watson–Crick base-pairing of nucleotides so that each polynucleotide chain can act as the template for another. Hydrogen bonds hold the base pairs, A-U and C-G together.

**Figure 10:**
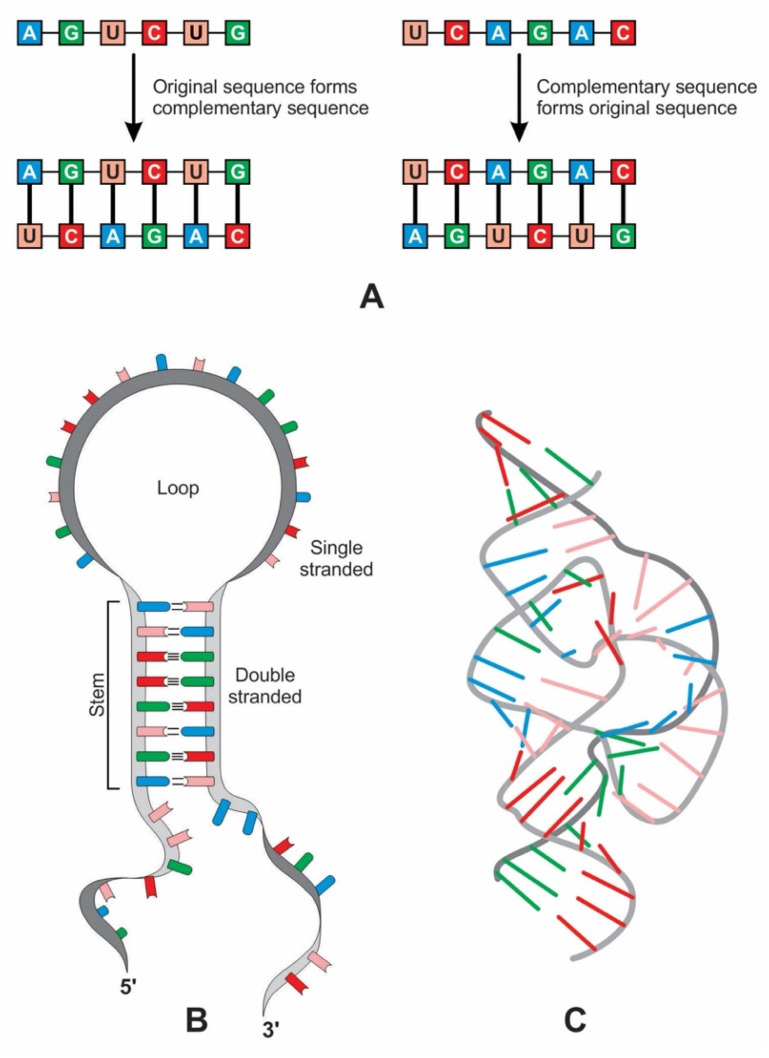
(**A**) replication of an RNA molecule by base-pairing. Left: the original RNA strand acts as a template to make a complementary strand by base-pairing. Right: this complementary RNA strand itself acts as a template, forming an RNA strand of the original sequence. (**B**) Although RNA is a single-stranded molecule, it can form a secondary hairpin structure of ribozyme. (**C**) Hammerhead ribozyme, like protein, can create tertiary structures and catalyze reactions; the tertiary structure can have both Watson–Crick and non-canonical base pairs.

Complementary base pairing, also known as ‘hybridization’, allows one RNA molecule to act as the template for another to form (Figure 10A). After replication, two double strands of RNA split into four single-stranded molecules, one of which is identical to the original strand. A single strand of RNA can specify a complementary polynucleotide sequence, a ‘flipped’ version of the original, while the second round of copying restores the original sequence [38].

Such a complementary mechanism producing more diverse populations of molecules lies at the heart of RNA replication. The non-enzymatic RNA-templated replication processes are always prone to errors. For years, researchers have questioned whether there might have been a simpler way to copy RNA—perhaps by polymerase ribozymes, but it remains highly speculative. Recently, an RNA polymerase in vitro evolution has shown promises that it can copy its own template with low fidelity [39]. Most likely, peptides assisted RNA replication inside protocells (Figure 7). RNA replication played a critical role in the base-pairing between codon and anticodon, giving rise to mRNA and genetic code, respectively.

### 6.2. The Origin of Ribozyme

Ribozymes are RNA molecules that can catalyze specific biochemical reactions in a way like protein enzymes. However, the primary structure of RNA molecules is much more restricted than that of proteins by having only four bases versus the 20 types of amino acids at the base of proteins. RNA can bend back on itself to form localized double-stranded regions in hairpin loops, resulting in a secondary structure (Figure 10B). It can also fold into various complex tertiary structures, three-dimensional structures that give ribozymes their catalytic ability (Figure 10C). Many ribozymes configure either a hairpin- or hammerhead-shaped active center. All ribozymes catalyze the cleavage of RNA chains or the formation of bonds between RNA strands. The hairpin structure of ribozyme is a key to many RNA secondary structures, such as pre-tRNA, bridge peptide, and ribosome. An RNA template used complementarity and template in addition to protocellular information to enable base pairing and replication [39]. Functional stabilization of ribozymes requires short peptide molecules, which were available in the peptide/RNA world.

**Figure 11:**
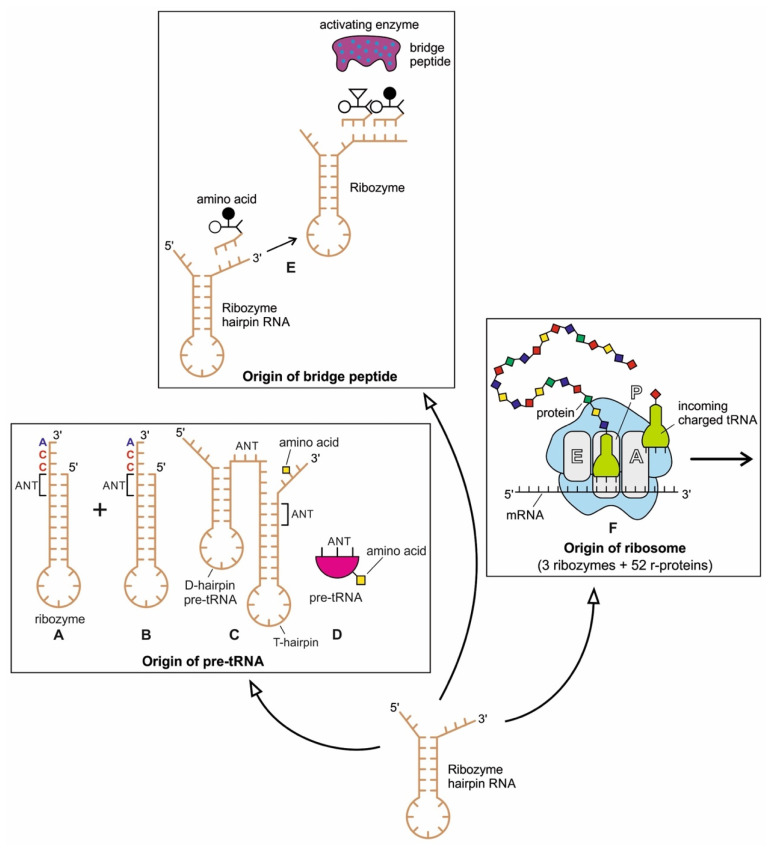
The origin of three components of translation machinery from the hairpin structure of ribozyme with a stem and loop: pre-tRNA molecules (**A**–**D**), bridge peptide (**E**), and ribosome (**F**). (**A**,**B**) The hairpin structure of two ribozymes, each with a loop and a stem. (**C**) The ligation or duplication of the hairpin structures may give rise to a double hairpin structure, forming a T-hairpin loop and D-hairpin loop with an anticodon (ANT) site between the two stems. (**D**) A schematic, simplified diagram of the pre-tRNA molecule showing the anticodon site and amino acid attachment site. (**E**) The hairpin ribozyme structure with a stem and loop and its activating enzyme, the bridge peptide. The amino acid is attached to its free oligonucleotide end by the bridge peptide. (**F**) Ribosome, a hybrid ribonucleoprotein complex, decodes the message of mRNA to synthesize a small protein chain. It is a decoder of digital information to analog information (modified from [40]).

The ribozymes play a central role in HIS and create translation machinery parts such as pre-tRNA, bridge peptides, and ribosome. In our previous paper [40], we discussed the likely scenario for the origin of a pre-tRNA molecule from the folded ribozyme with a stem and loop structure. The modern complex tRNA structure probably evolved from a simpler precursor, such as a pre-tRNA molecule (Figure 11A–D). Two ribozyme molecules with hairpin structures (stem and loop) probably created a pre-tRNA molecule by fusion or ligation [41,42].

The ribozyme also gave rise to ‘bridge peptide’, a precursor of pre-aaRS and aaRS enzymes (aminiacyl-tRNA synthetases) [40,43]. The ribozymes acquired amino acid at its 3′ end as a cofactor; an amino acid was attached to a ribozyme and made it a more efficient catalyst [44]. By using cofactors, the range of catalytic activity can be increased (Figure 11E).

Finally, ribozymes led to the origin of ribosomes (Figure 11F). Within the ribosome, ribozymes function as part of the large subunit ribosomal RNA to link amino acids during protein synthesis. Ribosome is fundamentally a peptidyl-transferase ribozyme supported by ribosomal proteins (r-proteins) that contribute to the correct folding of the RNA structure and improve the efficiency and accuracy of translation [45]. These startup molecules of translation machinery from ribozymes would continue to evolve for efficiency and functionality, as discussed below.

### 6.3. The Origin of tRNA Molecules

The tRNA molecule is short, typically 76 to 90 nucleotides in length. It serves as an adaptor molecule between mRNA and the amino acid sequences of proteins. It conveys the information contained in the nucleotide sequences of mRNA with the functional information contained in the proteins. The tRNAs would also create the first gene, as discussed later. Because of these critical roles, understanding the properties of tRNA molecules is critical in prebiotic information systems. Without tRNAs, genetics and coded protein synthesis are impossible. The primary structure and the overall geometry of tRNA molecules are undoubtedly more complex than those of any other RNA species.

**Figure 12:**
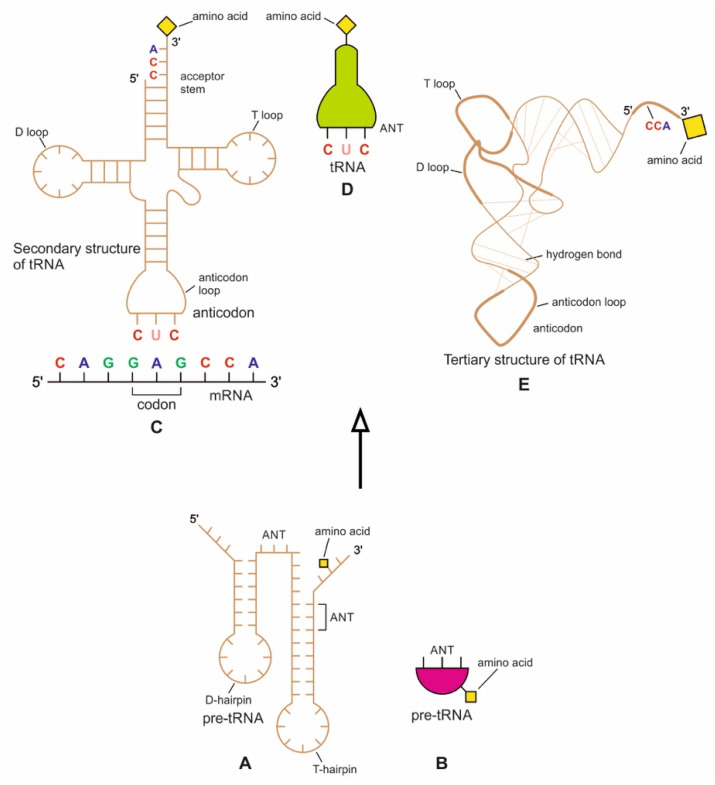
(**A**) The evolution of a tRNA molecule from a precursor pre-tRNA molecule (**A**,**B**) by gene duplication. (**C**) The secondary structure of a tRNA molecule could have been created by ligation of two half-sized pre-tRNA structures. Now a full-length tRNA structure looks like a cloverleaf; its anticodon end forms a complementary base pair with the codon of mRNA; (**D**) a simplified and schematic diagram of the tRNA molecule showing the site of the anticodon. (**E**) The cloverleaf secondary structure of tRNA could be folded into an L-shaped tertiary structure; it shows the aminoacylation site at the CCA end. The minihelix region (half domain of tRNA with the amino acid attachment site) interacts with the conserved domain of aaRS for amino acid activation. The other half of tRNA interacts with the non-conserved domain of aaRS for specific recognition of an anticodon (modified from [40]).

The origin of tRNA is contentious. Many studies suggested that the modern cloverleaf structure of tRNA may have arisen from a single ancestral gene by duplicating half-sized hairpin-like RNAs by passing through some intermediate structures such as pre-tRNA molecules (Figure 12A) [42,46,47]. The linkage of a ribozyme with the amino acid at the terminal of a hairpin loop might be the starting point for the origin of tRNA, a quarter the size of the modern tRNA molecule [46,47]. Pre-tRNA molecules, in turn, would give rise to tRNA molecules by structural rearrangement. The tRNA shows both secondary and tertiary structures (Figure 12C,E). The secondary structure of the tRNA molecule in the solution with three hairpin loops resembles a cloverleaf from nature (Figure 12C). One of these hairpin loops contains the anticodon, which forms base pairs with the codon of mRNA. The other two loops of the cloverleaf create a T-arm and a D-arm. The CCA sequence at the 3′ end of the acceptor stem forms a covalent bond to the amino acid that corresponds to the anticodon sequence. The CCA sequence of the acceptor stem offered a binding site for the amino acid. The 5′ terminal contains a phosphate group. The anticodon and the acceptor stem sequence correlate with amino acids’ role in folded proteins [47,48]. The secondary structure tRNA molecule may provide some clue as to its ancestral molecular configuration. The cloverleaf configuration of tRNA can be derived from a folded ribozyme with a single loop and an attachment site for the amino acid at the end of a stem.

The relevance of ribozymes in the origin of tRNA is enormous. The equivalent effect of gene duplication might be accomplished by a simple ligation of two identical hairpins of folded ribozymes to create double hairpins, a D-hairpin, and a T-hairpin, with an anticodon at the stem bases [47]. RNA ligation is a powerful driving force for the emergence of tRNA, joining two hairpin loops of the ribozyme (Figure 11C). During the evolutionary transitions of the pre-tRNA molecule, the double hairpin structure with the D-hairpin and the T-hairpin formed in the ancient prebiotic world anticodon in the terminal CCA sequence adjacent to the D-hairpin (Figure 11D) [49].

We suggest that this half-sized hairpin structure of the pre-tRNA molecule using anticodon and the amino acid binding site at the opposite ends acquired some functional capacity for translation before the emergence of tRNA (Figure 11A). In other words, pre-tRNA assists primitive protein synthesis using anticodon, which reads codon in pre-mRNA and the corresponding amino acid bound on CCA 3′ end. The pre-tRNA molecule is the evolutionary precursor of the tRNA molecule. Direct duplication or the ligation of half-sized, hairpin-like structures—the pre-tRNA molecule—could have formed the contemporary full-length tRNA molecules (Figure 11B). The acceptor stem bases and the anticodon stem/loop bases in tRNA 5′ half and 3′ half fit together with the double-hairpin folding; this suggests that the primordial double-hairpin RNA molecules could have evolved to the structure of modern tRNA by gene duplication, with subsequent mutations to form the familiar overleaf structure [49]. In other words, two pre-tRNA molecules somehow fused to form a tRNA molecule.

The half-sized pre-tRNA molecule with two loops (D-hairpin and T-hairpin) on one side, and anticodon and acceptor stem region of CCA end on the other side, is structurally and functionally independent and is more ancient than the other half of the tRNA molecule [41,42]. This short, self-structured strand of the pre-tRNA molecule possesses a template domain, which is chargeable through interaction with specific amino acids and is probably the predecessor of tRNA (Figure 11C). This pre-tRNA molecule binds, with high specificity, to the amino acid corresponding to its anticodon; this reaction is catalyzed by a specific pre-aminoacyl-tRNA synthetase (pre-aaRS). The tRNA evolution is closely linked to aminoacylation. There is a separate tRNA for each amino acid that carries a triplet sequence of nucleotides for anticodon. Later, the anticodon of pre-tRNA guides the codon formation of the pre-mRNA. The pre-tRNA and tRNA molecules became bilingual that recognized both the four-letter alphabet of nucleic acids and the 20-letter alphabet of amino acids. A ribozymal HIS developed in the form of a molecular distributed hybrid information system to use mechanisms such as RNA splicing, RNA cleavage, and peptide synthesis in various reactions.

### 6.4. The Origin of Bridge Peptides, Pre-aaRS, and aaRS Enzymes

Aminoacylation of tRNA is a crucial step in the translation system. In modern cells, enzymes perform the role of tRNA aminoacylation. Before the advent of protein enzymes, tRNA aminoacylation was probably carried out by the bridge peptide [40,43]. If it attaches an amino acid to its end, it would not be logical that the substrate amino acid is the cofactor simultaneously. This attachment first occurred to make cofactors, and ribozymes carried it. The ribozyme is performed as an assignment enzyme to bind a particular amino acid to an ancestral tRNA for aminoacylation before the emergence of aaRS [44]. We modified this view and suggested that another molecule available in the RNA/peptide world, namely bridge peptide, probably performed the function of an aminoacylation catalyst for the ligation of the amino acid with ancestral tRNA [40,42].

In the stem-loop configuration of a ribozyme, two ends of the stem might remain free, containing the 3′ and 5′ ends. This 3′ end might perform as an acceptor stem to form a covalent bond to a specific amino acid (Figure 12). This small hairpin ribozyme molecule with specific terminal base sequences acquired the corresponding amino acid as a cofactor to improve the catalytic range and efficiency [44].

Any specific binding between two molecules involves information sharing as if two molecules recognize each other. An amino acid can be linked to an oligonucleotide with three bases by an activating enzyme; the charged oligonucleotide is bound on a ribozyme’s surface by base pairing and delivers the appropriate amino acid (Figure 12). In this way, ribozymes can produce a short, de novo peptide chain that would play a role in stabilization to become coded. These peptide-forming ribozymes would function as amino acid-specific adaptors. In the peptide/RNA world, different kinds of peptides were synthesized. The availability of amino acids in the prebiotic environment would govern the aminoacylation of ribozymes. Aminoacyl-tRNA synthetases (aaRSs) are critical for the translational process, catalyzing the attachment of specific amino acids to their cognate tRNAs. The aaRS can recognize both amino acids and their corresponding anticodon of a tRNA. Interestingly, each aaRS recognizes all the tRNAs of the given amino acid. Therefore, it has imprinted in its structure one line in the genetic dictionary in cryptic form, with all synonyms included. There are 20 such enzymes, one for each acid [4]. Most likely, aaRS transferred this memory of amino acid-anticodon mapping to tRNA. The bridge peptide, a precursor to the aminoacyl transfer tRNA functioned as an activating enzyme supporting a primitive translation [44]. A bridge–peptide–aaRS complex HIS learned intermolecular hybridization and domain formation mechanisms to enable primitive translation. In our previous paper [40], we discussed how the bridge peptide might have given rise to protozyme to urzyme to pre-aaRS to aaRS step by step with the improvement of catalytic functions following the rule of continuity [50].

### 6.5. The Origin of Ribosome

We considered how ribozymes might have given rise to pre-tRNA and bridge peptides, the latter in turn to pre-aaRS to build up the primitive translation machine [40]. However, a translation machine needs one critical molecular machine, a ribosome, to read the message from mRNA continuously and make protein. Ribosomes are among the largest and most complex but elegant molecular nanobots. They are required for the genetic translation of mRNA and linking amino acids into a protein chain. In collaboration with the ribosome, the tRNA molecule helps decode an mRNA sequence into a protein chain (Figure 11F).

Most likely, two separate functions of ribosomes evolved simultaneously by accretion growth, the decoding functions in the small subunit and the peptidyl transferase center in the large subunit. The availability of simple proteins could have significantly enlarged the otherwise limited catalytic functions of the ribozyme. The ribosome might have first originated as a ribozyme that only later evolved for structural complexity when ribosomal proteins began to appear in primitive translation. These r-proteins stabilized the structure and complexity of evolving ribosomes and interacted with many rRNA sequences. Both the assembly and synthesis of ribosomal components must occur in a highly coordinated symbiotic system [46,47,51]. In our previous paper, we argued the likely origin of the ribosome [40].

The ribosome manifests the beautiful cooperation of two polymers, RNAs and proteins. A ribosome is a hybrid machine composed of one-third protein and two-thirds RNA. In this elegant machine, about 50 ribosomal proteins (r-protein) are wrapped up with four ribosomal RNAs (rRNA). A ribosome is, therefore, a ribonucleoprotein (Figure 11F). The rRNAs contribute to more than half of the ribosome’s mass.

Two major parts, the large (50S) and the small (30S) subunits, make up the ribosome (Figure 11F). The small subunit (SSU) decodes mRNA and reads the genetic code, and the large subunit (LSU) has a catalytic function with peptidyl transferase activity to synthesize a protein chain from the amino acids. In bacterial ribosomes, the small subunit consists of one ribosomal RNA and 21 ribosomal proteins, while the large subunit comprises two ribosomal RNAs and 31 ribosomal proteins. These two subunits fit loosely in a slot so that the ribosome can glide through the mRNA chain from 5′ to 3′ direction to read the encoded message in codons for the synthesis of protein. The output of the ribosome along with its embedded ribosomal HIS is the synthesis of the protein chain, which is a three-dimensional analog information system. Thus, the ribosome can be thought of as an efficient digital-to-analog converter.

Ribosomal RNAs are mainly hybrid components, but they function as catalysts like proteins. Thus, ribosomes perform two critical functions in prebiotic synthesis: (1) translate encoded information in mRNA to amino acids (2) and link together amino acids. The embedded ribosomal HIS is a distributed network of subunits and macromolecules that perform the ribosomal function.

The ribosomal RNAs are programmed to recognize the pairing between mRNA codons and complementary tRNA anticodons to a growing polypeptide chain (Figure 11F). When specific protein production is complete, the two subunits of the ribosome become separated and are recycled after each round of translation. Similarly, once the protein is made, mRNA is broken down, and the nucleotides are recycled. A ribosome is a complex hybrid nanobot that integrates analog and hybrid information systems and communicates between them.

### 6.6. Summary of Hybrid Information System

We discussed three critical molecular components of the translation machine: pre-tRNA/tRNA, pre-aaRS/aaRS, and finally, ribosome that would work in a complex repertoire to decode the digital information embedded in the nucleotide sequences of mRNA for the synthesis of protein. All the components of translation machines are bilingual and orchestrated nicely like an elaborate factory production line during the manufacturing of proteins. Once the translation machinery was in place, digital information could enter the system.

**Figure 13:**
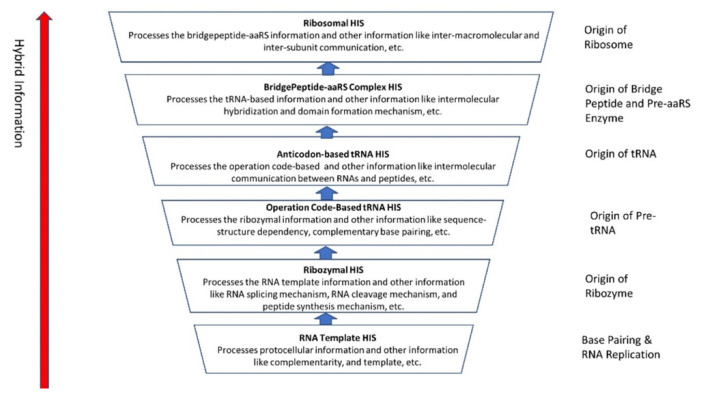
Hierarchical emergence of Hybrid Information System (HIS) during the early stage of peptide/RNA world. The most basic HIS is termed ‘RNA Template HIS’. The higher level (stage) HIS is built upon the lower level HIS. For example, the next stage HIS, the Ribozymal HIS, subsumes the RNA template HIS and so on.

Hybrid information systems started to take advantage of digital computing with qualities such as accuracy, memory, accurate replication, and faithful transmission by combining it with analog computing. Every biological system is made up of two types of interconnected parts—an analog part that processes analog quantities and interacts with the world and a digital part that processes digital quantities and helps preserve and reproduce itself. In the early stages of the biological information system evolution, the analog part of the system had become highly evolved through the processes of self-assembly of lipid chain into bilayer membrane, polymerization of monomers, encapsulation of polymers, insertion of peptides into lipid membrane, proto-metabolism, and growth and division of protocells. However, the digital part was random and ‘weak’. The digital part had to be given a more sustainable and reproducible form to have a more reliable and robust transfer of information horizontally and vertically. The earlier hybrid information systems were analog-oriented hybrid information systems. Digital computing has become a dominant component in a digital-oriented hybrid information system. A balanced hybrid information system makes efficient use of different types of analog and digital components. Figure 13 shows a hierarchy of hybrid information system (HIS) stages for the peptide/RNA World. RNA templates contain information in the digital (coded) form. Over the years, these templates were refined along with the development of increasingly robust and complex hybrid systems such as ribozyme, tRNA, bridge–peptide–aaRS complex, and ribosomal HIS.

## 7. Digital Revolution: Genes in the Making

The passage from HIS to DIS was smooth in the peptide/RNA world. HIS facilitated the emergence of hierarchical translation machines, which helped to coevolve the genome content of mRNA molecules. Barbieri [8] suggested that life is artifact-making and digital information plays a significant role in manufacturing artifacts. In the peptide/RNA world, DIS helped manufacture proteins by translation machines.

Because RNA contains four nucleotide bases analogous to the words’ letters, it can function as an information-containing molecule. However, the linear sequence of nucleotides in the early stage of abiotic RNA is like a nonsense word without much meaning. It does not follow any grammar or rules. It could not have specified anything and could not be said to carry much information other than the Watson–Crick base-pair rule. The most crucial task in the digital world was to assemble mRNA molecules step-by-step, creating codons and encoding these molecules with cognate amino acids by tRNAs for protein synthesis.

Along with the evolution of mRNA, translation and the genetic code coevolved with DIS. Side by side, three generations of translation machines in HIS and three generations of proteins in AIS evolved hierarchically. Directionality appeared in three types of information systems from DIS to HIS to AIS to complete this cycle.

### 7.1. Selection of Amino Acids

Seventy amino acids were identified in the Murchison meteorite [19,20]. Miller’s experiments created more than 40 different amino acids [52]. Despite the availability of many amino acids in the prebiotic environment, nature has selected twenty amino acids in translation. It is likely that twenty amino acids needed for protein synthesis were not available initially; they were selected step-by-step from a small number of abiotic simple amino acids; these precursors gave rise to more complex amino acids. Wong [53] championed the coevolution theory, and Di Giulio [54] further expanded this model. It proposes that the genetic code is an imprint of the biosynthetic relationships between amino acids. The primitive proteins were created only by those 10 amino acids that were readily obtainable from the prebiotic environment. The remaining 10 amino acids entered the prebiotic system as the code expanded to the universal code.

Ikehara [55,56] proposed the GADV hypothesis that four primitive amino acids, glycine (G), alanine (A), aspartic acid (D), and valine (V), were available in the prebiotic soup. We suggest that these four amino acids would govern the emergence of four codons (GGC, GCC, GAC, and GUC) by corresponding pre-tRNAs. Because pre-aaRS could recognize both amino acid and its corresponding anticodon of a pre-tRNA molecule, these GADV amino acids would be selected by pre-aaRS enzymes to charge cognate pre-tRNA molecules. These four pre-tRNAs, in turn, would create corresponding codons by base pairing, thus initiating the codon–amino acid mapping.

### 7.2. Processing and Assembly of mRNAs: The Emergence of Genes

In DIS, we see the beginning of creating the coding RNA (mRNA), which was designed and custom-made by a noncoding RNA (tRNA). The RNA molecules that initiated protein synthesis belonged to messenger RNA. The mRNA is the most critical molecule for the origin of DIS, but its birth in the peptide/RNA world remains unknown. In living cells, mRNA is directly transcripted from DNA, where an enzyme (RNA polymerase) converts the gene of a DNA chain into the primary transcript mRNA. However, how did mRNA emerge before the advent of DNA in the peptide/RNA world where there was neither protein enzyme nor DNA? Here we propose a model for the origin of the first genes before transcription.

There would have been various mechanisms of non-enzymatic polymerization of nucleotides into RNA (e.g., by a condensation reaction in which phosphodiester bonds are formed). However, these abiotic RNAs would have been noncoding RNAs, and they lacked the genetic triplet code for protein synthesis.

The tRNA is a key and mobile molecule of the translation machine. It matches with the codons and ferries correct amino acids to the ribosome. It is an adaptor molecule between codon and amino acid. In our model, it played a key role in the prebiotic synthesis of mRNA. Here we suggest a likely biochemical pathway for the origin of coded mRNAs, which would direct protein synthesis. tRNAs would create codons in mRNA in two successive steps: (1) creating codons by anticodons by base pairing, and (2) encoding these codons by charged tRNAs, transferring information of cognate amino acids. We discuss later how these newly formed codons would be encoded by tRNA containing specific information about amino acids. Codon recognition describes the process of matching codons to the correct amino acids.

### 7.3. Piecemeal Buildup of Codon Sequences in Pre-mRNA by Pre-tRNA Molecules

**Figure 14:**
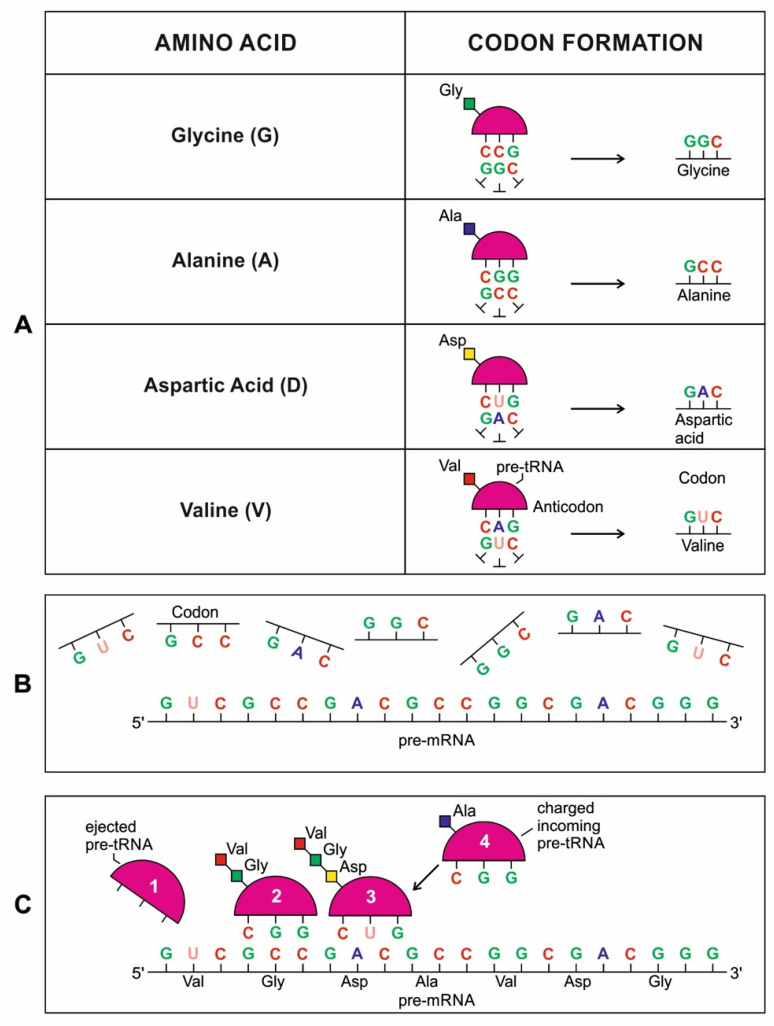
Creation of codons by pre-tRNA molecules step by step. (**A**) GADV amino acids govern the origin of codons via pre-tRNAs; anticodon of a pre-tRNA molecule hybridizing with the corresponding nucleotide available in the prebiotic soup to form a codon strand; each codon developed a memory for a specific amino acid. The four amino acids, glycine (G), alanine (A), aspartic acid (D), and valine (V), were available in the abiotic stage. (**B**) Codons, thus created by pre-tRNAs, began to link to form a strand of pre-mRNA with coding sequence; (**C**) Pre-tRNA and pre-mRNA interactions generated rudimentary translation. In this figure, we offer a specific mapping mechanism between codons and their cognate amino acids that led to rudimentary translation and the genetic code (modified from [40]).

Codons are the three-letter snippets of mRNA that provide instructions for the specific arrangement of amino acids, which are the building blocks of making proteins. Encoding of pre-mRNAs could be created by pre-tRNAs step-by-step by linking these codons into longer chains. We begin with the pre-tRNAs that might have designed the codons of pre-mRNA molecules for storing digital information. As the pre-tRNA molecules begin to map specific amino acids, they need a separate storage device for the preservation of amino acid information. Pre-tRNAs begin to create custom-made ancestral pre-mRNA strands for the safekeeping of their amino acid information. These pre-mRNA molecules began to map primitive groups of GADV amino acids such as glycine, valine, aspartic acid, and alanine that they tend to encode (Figure 14A).

In our model, pre-tRNA molecules charged with specific amino acids began to select nucleotides from the prebiotic soup via base-pairing with their anticodons; these nucleotides were joined to form a codon strand with memory for a specific amino acid, transmitted by the anticodon of a pre-tRNA. The short codon segments, in turn, are polymerized to create a long strand of pre-mRNA for storing digital information about amino acids.

### 7.4. Random Linking of Codons and Polycodons to Pre-mRNA Chain

Similar to nucleotides, codons could be linked to one another in a chain by a condensation reaction to form oligonucleotides and pre-mRNA-like molecules. Olasagasti and Rajamani [57] showed the polymerization of RNA-like molecules experimentally from non-activated prebiotic mononucleotides and oligonucleotides via condensation reactions. These authors simulated a terrestrial hydrothermal environment that fluctuates between wet and dry cycling during seasonal change. They showed that a mixture of lipids and mononucleotides or oligonucleotides could produce relatively long strands of RNA-like polymers in alternate cycles of dehydration and hydration. It is likely that in a hydrothermal crater vent environment, newly formed codons could link together to pre-mRNA-like molecules by a hydration–dehydration cycle. Clay mineral particles have long been known to have large adsorption capacity and the ability to concentrate and polymerize monomers [22]. The catalytic power of clay minerals can stimulate the polymerization and linking of codons at the floor of the hydrothermal crater basin. The linking of codons could be achieved on the mineral substrate either by the hydration–dehydration cycle at the sloping rim of the crater basin or the floor of the crater basin (Figure 2). Although four codons were formed initially by anticodons of pre-tRNA molecules, 24 codons could be generated by combinations of each of these four codons that could create six polycodon chains. These six polycodons, in turn, could generate 720 polycodons by combinations and link randomly to the form of pre-mRNA molecules (Figure 14B,C). The wet/dry cycle at the hydrothermal crater edge was a natural experimental laboratory for the random assembly of codons and polycodons into a pre-mRNA chain. This pre-mRNA became the binding partner for pre-tRNA, enhancing mutual stability and instant recognition.

As pre-tRNAs created more and more codons by base-pairing, the longer chain of pre-mRNA and mRNA would be created to store genetic information for the synthesis of long and complex protein molecules. The origin of pre-mRNA and mRNA was the first important step of digital information during life synthesis.

### 7.5. Encoding the Message

Amino acids do not read their codons. Similarly, codons and amino acids do not recognize each other directly but use molecular intermediaries. The protein and mRNA languages seem unrelated. The mRNA stores the amino acid information to assemble proteins using the four alphabets A, U, C, and G. In contrast, proteins employ twenty different sorts of amino acids. Therefore, how do mRNAs and proteins communicate? How, then, is the message read? The bilingual tRNA acted as an interpreter and helped to read the message of pre-mRNA, a sequence of codons, to the language of a protein, a sequence of amino acids. Nature has discovered a neat solution to the numerical mismatch by packaging the bases in triplet codons. Four bases made of three nucleotide triplets or codons can be arranged in sixty-four (4^3^) possibilities of codons to code 20 amino acids, and three codons are used as stop signals. Consequently, the universal genetic code is redundant or degenerate because more than one codon may code a single amino acid. The genetic code is nonoverlapping, meaning a single nucleotide cannot share two adjacent codons.

The genetic code was deciphered nearly 60 years ago. Despite decades of effort, it remains largely unknown why certain amino acids are assigned to certain codons. Why do the codons encode specific amino acids during protein synthesis? This is one of the most perplexing questions in molecular biology. There is no direct codon/amino acid interaction during translation, but anticodon preserves its information of cognate amino acid. Each tRNA molecule has two functional domains: the acceptor domain for bonding and recognizing its cognate amino acid and the second anticodon domain for preserving the memory of its specific amino acid. In aminoacyl tRNA (or charged tRNA), its cognate amino acid is chemically bonded, corresponding to its anticodon. A set of aaRS enzymes catalyzes the charging of tRNA. No such chemical bond exists between the codon in mRNA and its assigned amino acid. The existence of bilingual adaptors such as charged tRNAs may provide insights into the codon–amino acid association. Here we offer a likely mechanism of encoding codons by tRNAs. Charged RNAs might function as the donor of amino acid memory to corresponding codons in mRNA.

### 7.6. Encoding Properties of Pre-tRNA and tRNA Molecules

Pre-mRNAs would give rise to mRNAs by gene duplication or linking several strands of pre-mRNA. Recycled mRNA molecules after the translation were an essential activity and essential for replenishing the nucleotide pool to create a new mRNA chain that would enable a protocell to change its protein expression. Through many generations, natural selection would create encoded mRNAs that were programmed for protein synthesis without the assistance of tRNAs.

**Figure 15:**
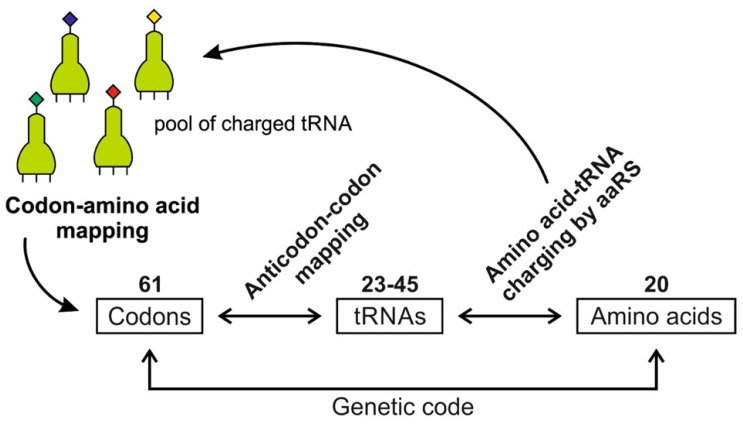
The encoding properties of tRNA. tRNA played two critical roles in creating and encoding codons corresponding to two different genetic codes. First, it created a codon by Watson–Crick base pair interaction (anticodon–codon mapping). Secondly, each charged tRNA transferred its amino acid information to the corresponding codon (codon–amino acid mapping). Participation of aaRS in the recognition process is an attractive possibility.

According to their two different genetic codes, pre-tRNAs and tRNAs have two distinct properties [58,59]. The accuracy of encoding depends on the precision of the two successive independent matchings. The first matching is manifested in the anticodon of tRNA that created uncoded codon by Watson–Crick base pair interaction. We call this stage *anticodon*–*codon mapping*. We used anticodons of tRNA molecules to develop an uncoded sequence of codons, analogous to a blank tape in a tape recorder. Just like how a magnetizable coating is placed on a plastic film to prepare it for storing musical data, the uncoded sequence of codons is prepared to place amino acid information in the second matching. The second matching binds each tRNA with its cognate amino acid in relation to its anticodon, and the charging is catalyzed by specific aaRS. There are twenty such aaRSs, one for each amino acid. Each charged codon then moves its amino acid information to its corresponding codon. We call this second information stage *codon*–*amino acid mapping (*Figure 15). The second code is written in the structure of aaRS and is the likely mechanism of encoding codons with amino acids.

The two-step encoding mechanism of codons by tRNAs left a detectable imprint in stop codons. The three stop codons (UAA, UAG, and UGA) signaling translation termination may signify that these three codons are relics of the first anticodon–codon mapping without amino acid input.

We used the codon–amino acid mapping scheme to place specific amino acids into their corresponding codons. The blank tape of pre-mRNA and mRNA becomes a recorded tape, mapping each codon with its specific amino acid. Our model in Figure 15 shows a viable method of encoding codons by tRNAs. These codons remember that they code amino acids and store digital information and become memory tape. These encoded codons develop molecular memory. After mRNA translation into a protein chain, mRNA molecules are recycled into nucleotides. These nucleotides, when polymerized to form a new mRNA chain, inherit this memory, and become encoded permanently by natural selection. The tRNAs became the matchmakers between codons and their corresponding amino acids. However, codons cannot attach chemically to appropriate amino acids. Moreover, they can store this digital information of amino acids transferred by tRNAs in their molecular memory like a tape recorder. The tRNA was a molecular architect that designed and encoded mRNA. If tRNA did not create encoded mRNA, then its bilingual ability to read nucleotide sequences of mRNA and process amino acid information for protein synthesis is difficult to explain. tRNA transferred amino acid knowledge from a source domain to a target domain such as uncoded codons as in a machine learning system.

In recent years, artificial intelligence programs have reached a surprising level of linguistic fluency, where the learning algorithm is called neurons. Prebiotic information system developed the language processing by natural selection four billion years ago, which is still operating in all living organisms with surprising accuracy and fidelity. Like machine language, we propose a progressive *memory bank* for incremental domain adaptation in codons in three stages of the genetic code. A memory bank is storage of translation memory in a neural network for machine translation [60]. The mRNA can be viewed as a mobile storage device for storing amino acid information in its codon sequences. Today, encoded mRNA is transcripted and directed from DNA, but in the peptide/RNA world, our model may provide an insight into how the ancestral genes might have evolved before transcription when genetic memory developed in pre-mRNA and mRNA in the linear sequences of codons. The genetic code evolved as a progressive memory bank for codons to remember their amino assignments, and the memory is retained permanently by the natural section. The correspondence between a codon and an amino acid was indirectly realized through amino acid information transfer by tRNA. The memory bank concept may solve the riddle of the origin of the genetic code. The transfer of two sets of information from tRNA to mRNA was abandoned and erased from the living digital information system in molecular biology when DNA took over.

### 7.7. The Beginnings of Translation and the Genetic Code

It follows from the above discussion that the actual step of translation from mRNA or genes into protein language occurs when anticodons of charged tRNAs are matched with codons of encoded mRNAs. The translators are charged tRNAs. If the creation and encoding of codons are molecular rehearsals, the translation is the actual acting. Codon recognition became easy in translation. The two processes of tRNA codes replay during mRNA translation in two successive independent matchings. First, the anticodon of tRNA detects the codons by base pairing; it reads the message according to the genetic code. Second, each charged tRNAs ferries cognate amino acids to the ribosome and is linked together into a polypeptide chain as the mRNA passes through and is ‘read’ by the ribosome.

The genetic code defines a mapping between codons and amino acids. The mapping of the codon to its corresponding amino acid evolved with time. In the primitive stage (GNC code), four codons encoded four specific amino acids, glycine (G), alanine (A), aspartic acid (D), and valine (V), which is aptly called the GADV hypothesis [53,54]. The molecular encoding of four codons with their cognate amino acids was embedded by pre-tRNA by innumerable frequencies in the vent environment and is somewhat analogous to machine learning of a bilingual language (e.g., English to German) that lets computers learn to program themselves through experience. In this case, the program is the GNC code. Codon GGC representing glycine (G), codon GCC for alanine (A), codon GAC for aspartic acid (D), and GUC representing valine (V) became permanently embedded in the genetic memory of these codons (Figure 14A). Genetic memory between codon and its cognate amino acid is formed by a sequence of repeated interactions in the vent environment, where the process has a starting point, goes through a sequence of steps, and has an endpoint. Then the cycle starts over again. Each process was able to ‘remember’ its steps and the memory needed to complete the process. These codons ‘remembered’ which information of amino acids they code and store. This is how genetic memory developed in mRNA that resides in the linear codon sequences. They may be regarded as the memory mapping of the polypeptide structure [11].

This process of genetic memory continued to enrich as more molecular data were available in the vent environment. In the next stage (SNS code), 16 codons encoded ten amino acids with the introduction of redundancy so that several codons represented the same single amino acid, but there were no ambiguities. There were no examples of a single codon representing more than one amino acid [54,55,56]. Among these 16 codons, 12 new codons were embedded with amino acid information by tRNAs. The early genetic code continued to evolve.

In the final stage (universal genetic code), 64 codons represented 20 amino acids with a significant component of redundancy. Of these, three codons do not specify amino acids. Out of 61 codons, 45 new codons encoded amino acid information, assisted by tRNA molecules, but three remained uncoded and functioned as stop signals. These stop codons tell when a polypeptide is complete. The tRNA has imprinted one line of the genetic dictionary in mRNAs, with all synonyms included. With stable memory sets in 61 codons for amino acid information and an efficient translation mechanism established by countless natural experiments in the vent environment, the universal genetic code started operating with high fidelity in the peptide/RNA world. The mapping between 64 codons and 20 amino acids created a permanent memory bank, which became the universal genetic code by natural selection.

The design of custom-made pre-mRNA by pre-tRNA was a watershed event in the origin of translation when a pre-mRNA molecule becomes a storage molecule for genetic information in a separate digital device. Eventually, several strands of pre-mRNA are linked to form a longer strand of pre-mRNA, about 30 to 80 nucleotides. In the prebiotic soup of the hydrothermal vent environment, varied lengths of pre-mRNA strands by permutation and combination evolved, storing and encoding a wide range of amino acid information to synthesize longer protein chains.

### 7.8. Summary of Digital Information System

**Figure 16:**
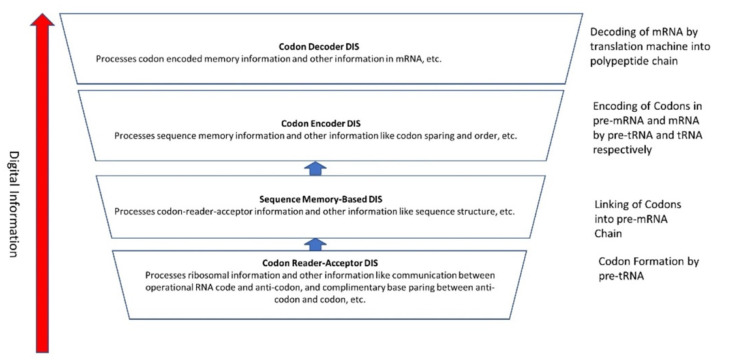
A four-level hierarchy of Digital Information System (DIS) stages in the peptide/RNA world. The codon reader-acceptor DIS was able to form a codon. The sequence, a memory-based DIS in the next stage, was able to link codons into pre-mRNAs. The codons in pre-mRNA and mRNA were encoded by pre-tRNA and tRNA, respectively. Finally, mRNA was decoded by translation machine to create protein chain.

We divide the DIS into two parts: the first part deals with the making of mRNA by tRNA, the first gene, and the second part, which is well-known in the literature, is the origin of the genetic code. This section discusses the first part of how codons in pre-mRNA and mRNA were created and encoded by pre-tRNA and tRNA molecules with information on cognate amino acids. Without the origin of pre-mRNAs and mRNAs, translation and genetic code would not occur. Codons of pre-mRNA and mRNA learned how to store and transmit information in digital form. The encoded genetic and other biological information in digital form enables a system to store, reproduce, and transmit information precisely and efficiently. As noted above, a digital form of information is more stable and less prone to noise and degradation. This form became a natural choice for storing and transmitting information faithfully (Figure 16).

Pre-tRNA and tRNA played two critical roles in creating codons and encoding codons of pre-mRNA and mRNA by two distinct genetic codes. First, it created codons by anticodons using Watson–Crick base pair interaction (anticodon–codon mapping). Secondly, each charged tRNA transferred its amino acid information to the corresponding codon (codon–amino acid mapping). We showed these processes in simulation and visualization.

Hierarchically we identify four levels of DIS here: (1) codon formation by pre-tRNA, (2) linking of codons to pre-mRNA and RNA, (3) encoding of codons by pre-tRNA and tRNA, and (4) decoding mRNA into protein.

The culmination of the DIS is the decoding of an mRNA strand by the translation machine into a protein chain using the universal genetic code. In Section 9, we discussed the origin of the genetic code.

## 8. Simulation and Visualization of Encoding mRNA Molecules: The Origin of Genes

Here we used computer simulations to visualize the two-step processes of encoding mRNAs by tRNAs, integrating bioinformatics and biomolecular data in prebiotic synthesis to test our hypothesis (Figure 16). We simulated and visualized encoding processes of pre-mRNA and mRNA by using AnyLogic software (www.anylogic.com, accessed on 1 February 2022) at three stages of the evolution of the genetic code [40]:

- GNC code encoded by pre-tRNA/pre-aaRS machine;
- SNS code encoded by tRNA/aaRS machine;
- UG code encoded by tRNA/aaRS/ribosome nachine.

The universal genetic code translated 61 codons into 20 amino acids using 23 to 46 tRNA molecules, depending on the organism. There are fewer tRNAs than codons because of the ability of tRNA molecules to ‘wobble’ at the third base to decode more than one codon [61].

**Scheme 1:**
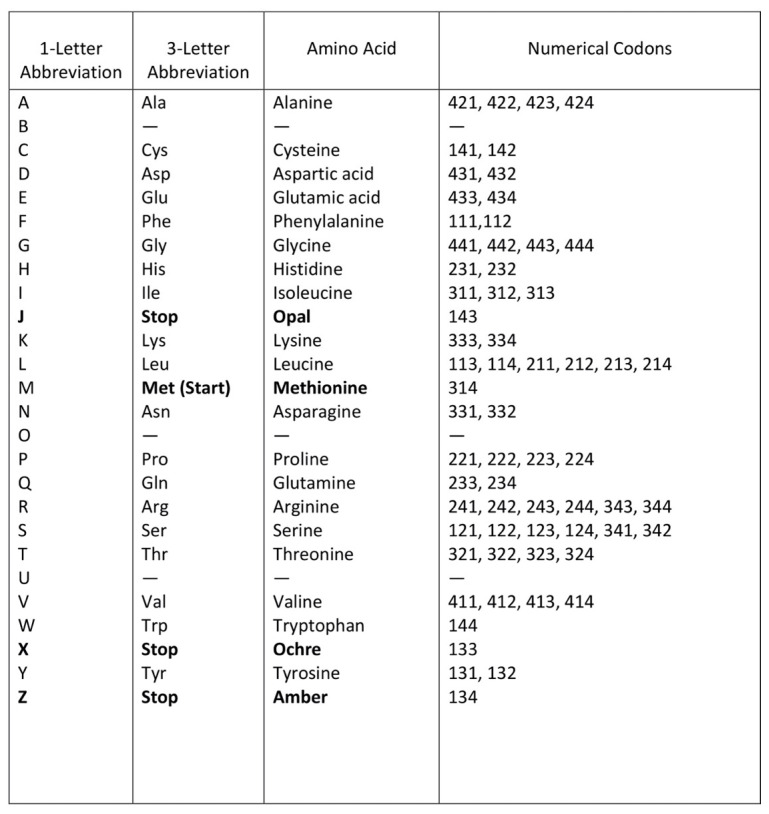
Twenty primary amino acids in the Genetic Code and their corresponding numerical codons shown by 23 alphabets. This represents the decoding table from mRNA to protein translation. The three letters B, O, and U remain unused.

We showed the amino acid–codon relationships for simulation: 20 amino acids in one-letter abbreviations corresponding to their codons in numerical forms. We substituted the nucleotide alphabets of mRNA with numbers as follows: 1 for U, 2 for C, 3 for A, and 4 for G [40] (Scheme 1). We used three additional letters, J, X, and Z (displayed in bold font), to signify three stop codons, namely opal, ochre, and amber, respectively.

**Scheme 2:**
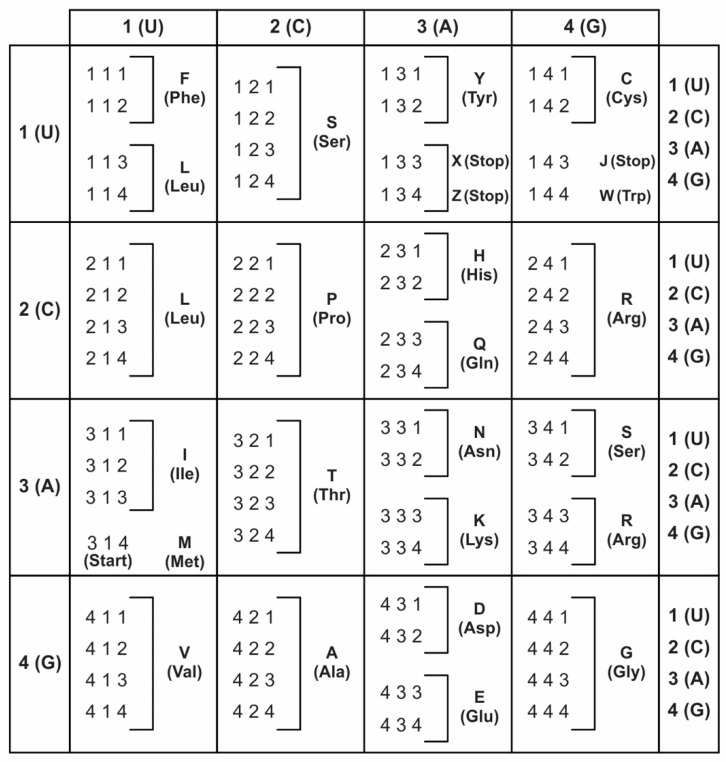
Universal Genetic code showing numerical codons with corresponding amino acids.

In Scheme 2, the abbreviation of the universal genetic code table is shown in numerical codons with redundancies. Each matrix cell displays information in both numerical codons and their corresponding amino acids. Because of the numerical distribution of codons in rows and columns, one can easily visualize the distribution of codons and their redundancies in the matrix cells, which was less evident in standard genetic code using combinations of four letters. Looking at Scheme 2, we can see those codons beginning with four formed first (the GNC code), followed by codons starting with 2 (the SNS code). Codons with prefixes 1 and 3 were added last to create the universal genetic code.

**Scheme 3:**
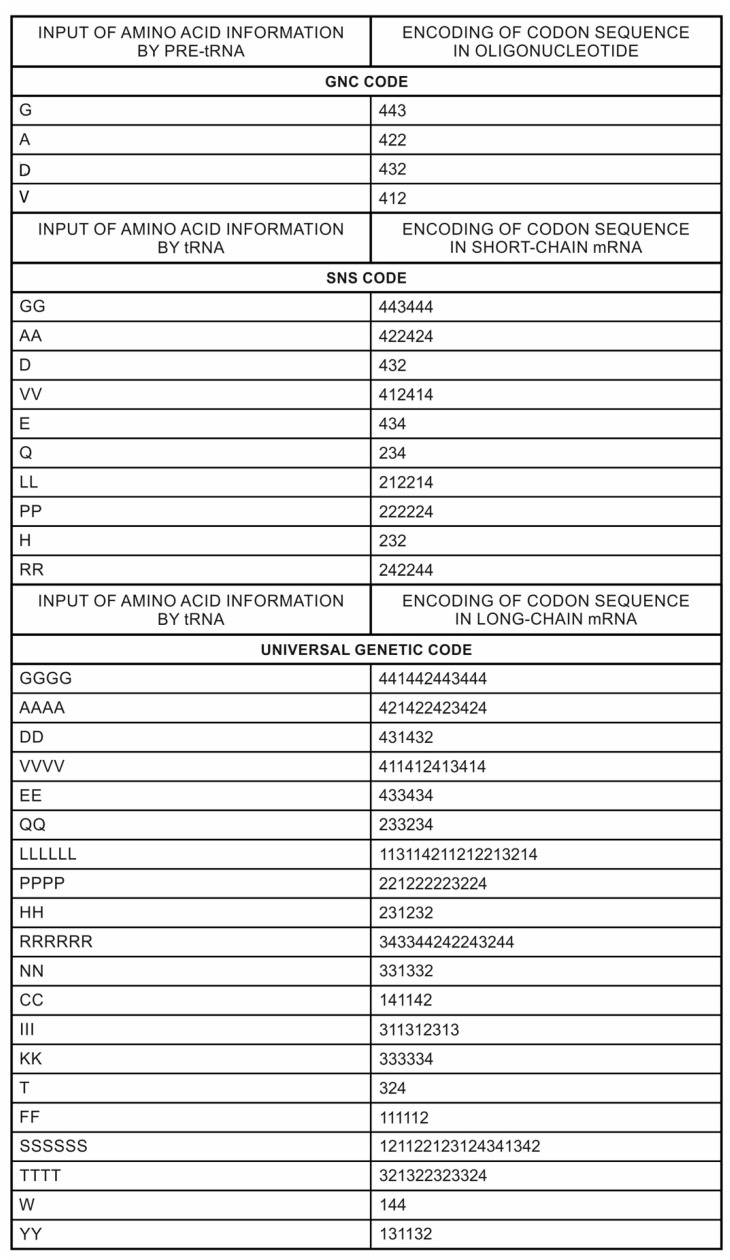
Codon–amino acid mapping in three stages of genetic code using CATI software. In SNS and universal genetic code, the sequence of generating redundancy of codons to amino acids is shown.

In our previous publication [40], we have developed a software called CATI (**C**odon–**A**mino Acid **T**ranslator **I**mitator) to translate numerical codons into corresponding one-letter abbreviations of amino acids and vice versa. Here, we used CATI to map the three stages of amino acid formation (letter) that code corresponding codons (in number) (Scheme 3).

**Figure 17:**
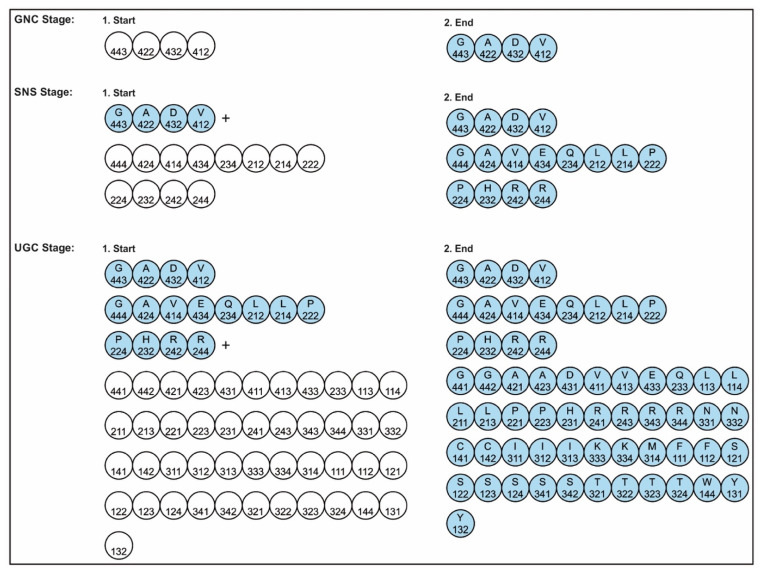
Codon–amino acid mapping and the origin of genes. Encoding codons by charged pre-tRNA and tRNA molecules in the three stages of the genetic code, controlled by the availability of amino acids in hydrothermal crater vent environment. In the GNC code, four pre-mRNA codons specify the four amino acids. In the SNS code, 16 mRNA codons code ten amino acids. In the universal genetic code, 61 mRNA codons designate the 20 amino acids. In the left column of each stage, the white circles represent the uncoded codons, while the blue codons represent encoded codons. Twenty-three to forty-five charged tRNA molecules perform the task of encoding codons.

In our visualization model, uncoded codons were already created by anticodons of pre-tRNA and tRNA by Watson–Crick base pair (anticodon–codon mapping) (see Figure 17). This is the first code of pre-tRNA/tRNA. Here we simulated and visualized the second code of pre-tRNA/tRNA and how these newly generated codons were encoded (codon–amino acid mapping). We showed these codon–amino acid mapping in the three stages of the evolution of the genetic code (Figure 17).

### 8.1. Stage 1. Visualization of Encoding Codons of Pre-mRNA by Pre-tRNAs in the GNC Code

In the start cycle of the GNC code, pre-tRNAs created four codons by hybridization: 443 (GGC), 422 (GCC), 432 (GAC), and 412 (GUC) (shown by white circles). In the end cycle, these codons were encoded by charged pre-tRNA, one at a time, with four cognate amino acids, including G (glycine), A (alanine), D (aspartic acid), and V (valine), respectively. This is often called the GADV model based on the availability of primordial amino acids [55,56].

### 8.2. Stage 2. Visualization of Encoding Codons of mRNA by tRNAs in the SNS Code

In the start cycle of the SNS code, four codons in blue circles were already encoded in the GNC code. Twelve white circles represent new additional codons that remain uncoded. These are: 444 (GGG), 424 (GCG), 414 (GUG), 434 (GAG), 234 (CAG), 212 (CUC), 214 (CUG), 222 (CCC), 224 (CCG), 232 (CAC), 242 (CGC), and 244 (CGG). In the end cycle, these 12 codons were encoded by charged tRNAs in the following order of corresponding amino acids: G (glycine), A (alanine), V (valine), E (glutamic acid), Q (glutamine), L (leucine), L (leucine), P (proline), P (proline), H (histidine), R (arginine), and R (arginine). Here we see the beginning of redundancy that some amino acids are coded by more than one mRNA codon. For example, the amino acid glycine is specified by the codons 443 and 444, and the amino acid alanine by 422 and 424 (see Scheme 3) [55,56].

### 8.3. Stage 3. Visualization of Encoding Codons of mRNA by tRNAs in the UG Code

In the start cycle of the universal genetic code, 16 codons in blue circles were encoded by the GNC and the SNS codes. Fourty-five white circles represent new additional codons that remain uncoded. These codons include: 441 (GGU), 442 (GGC), 421 (GCU), 423 (GCA), 431 (GAU), 411 (GUU), 413 (GUA), 433 (GAA), 233 (CAA), 113 (UUA), 114 (UUG), 211 (CUU), 213 (CUA), 221 (CCU), 223 (CCA), 231 (CAU), 241 (CGU), 243 (CGA), 343 (AGA), 344 (AGG), 331 (AAU), 332 (AAC), 141 (UGU), 142 (UGC), 311 (AUU), 312 (AUC), 313 (AUA), 333 (AAA), 334 (AAG),314 (AUG), 111 (UUU), 112 (UUC), 121 (UCU), 122 (UCC), 123 (UCA), 124 (UCG), 341 (AGU), 342 (AGC), 321 (ACU), 322 (ACC), 323 (ACA), 324 (ACG), 144 (UGG), 131 (UAU), and132 (UAC).

In the end cycle, these 45 codons were encoded by charged tRNAs in the following order of cognate amino acids: G (glycine), G (glycine), A (alanine), A (alanine), D (aspartic acid), V (valine), V (valine), E (glutamic acid), Q (glutamine), L (leucine), L (leucine), L (leucine), L (leucine), P (proline), P (proline), H (histidine), R (arginine), R (arginine), R (arginine), R (arginine), N (asparagine), N (asparagine), C (cysteine), C (cysteine), I (isoleucine), I (isoleucine), I (isoleucine), K (lysine), K (lysine), M (methionine), F (phenylalanine), F (phenylalanine), S (serine), S (serine), S (serine), S (serine), S (serine), S (serine), T (threonine), T (threonine), T (threonine), T (threonine), W (tryptophan), Y (tryosine), and Y (tryosine).

In the UC code, the redundancy is extreme, where 61 codons code 20 amino acids. For example, the amino acid leucine is specified by six codons UUA, UUG, CUU, CUC, CUA, and CUG.

A visualization model of the three stages of encoding codons of pre-mRNAs and mRNAs by pre-tRNA and tRNA molecules was created using AnyLogic software. The visualization model shows how the codons were encoded step-by-step by charged pre-tRNAs and RNAs in the prebiotic soup, mediated by pre-aaRS and aaRS enzymes. Each codon then developed permanent memory of its cognate amino acid by natural selection. The model shows the origin of genes step-by-step before transcription. Appendix A provides instructions on how to run the visualization model in the AnyLogic cloud.

## 9. The Origin of the Genetic Code

Even though the entire codon catalog was deciphered more than 60 years ago, the origin of the genetic code remains elusive. In our previous publication, we discussed in-depth the genetic code and offered a new model for its origin [40]. Here, we summarized some of the salient points about the origin of the genetic code considering the new information paradigm. In our view, genetic code evolved as a memory bank during the encoding of mRNA by tRNA molecules step-by-step, analogous to the memory bank of machine translation. Genetic code is the memory bank of codon–amino acid mapping. The mRNA-directed protein synthesis was the culmination of DIS in the peptide/RNA world, where HIS translation machine reads the message of the encoded mRNA of DIS to produce protein in the AIS format. Protein synthesis was guided by the rules of the genetic code.

### 9.1. Coevolution of the Translation Machine and the Genetic Code

During the evolution of the genetic code, DIS, HIS, and AIS are so intermingled that they worked in tandem: DIS for the enrichment of the genome content of mRNA, HIS for refinement of the translation machine, and AIS for the manufactured product of translation—protein synthesis. The unidirectional flow of information developed from DIS to HIS to AIS.

**Figure 18:**
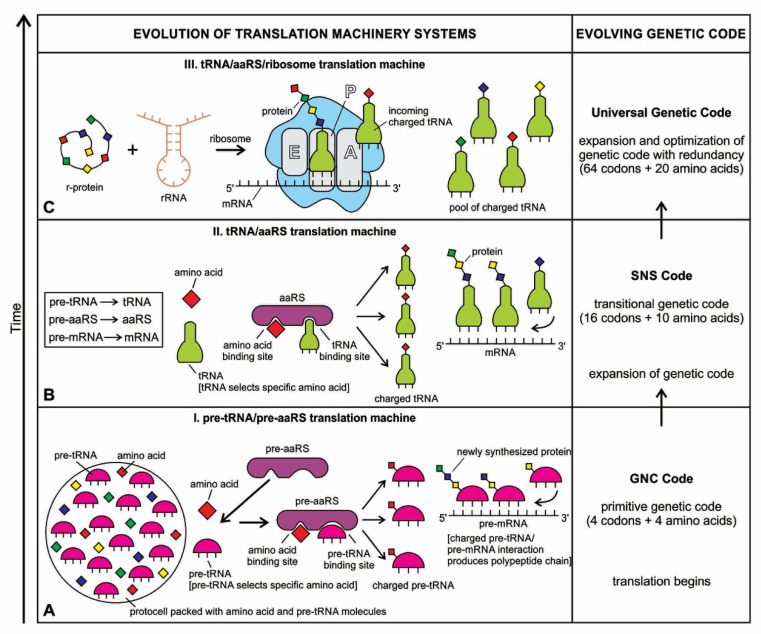
The coevolution of translation machines and the genetic code in three stages: (**A**) encoding of pre-mRNA molecule by pre-tRNA/pre-aaRS translation machine when GNC code evolved; (**B**) encoding of short-chain mRNA molecule by tRNA/aaRS translation machine when SNS code appeared; and finally, (**C**) encoding of long-chain mRNA by tRNA/aaRS/ribosome machine when universal code evolved. With the improvement of the translation machine, the information density of mRNA also increased (modified from [40]).

We suggest that three stages of the development of the genetic code proposed by earlier workers [54,55,56], which were coevolved with the concurrent improvement of the translation machines: the GNC code that was started by the pre-tRNA/pre-aaRS machine, the transitional SNS code progressed by the tRNA/aaRS machine, and finally, the universal genetic code blossomed by the tRNA/aaRS/ribosome machine [40]. The ribosome is the latest addition to the translation machine. It automatically joins an amino acid to a growing polypeptide chain. It is a small, dense particle constructed of a large and a small subunit, each made of RNA and protein components. Here, we identified three stages of the coevolution of the translation machine and the genetic code (Figure 18).

### 9.2. Decoding of Pre-mRNA by Pre-tRNA/Pre-aaRS Machine

**Figure 19:**
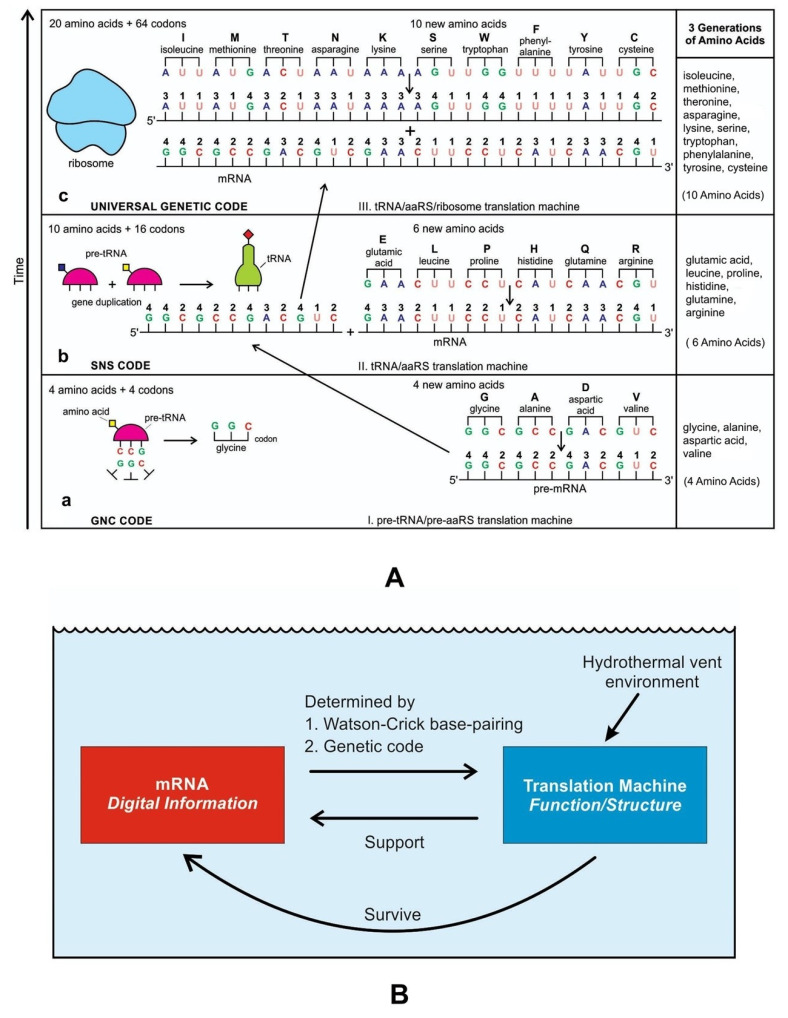
(**A**) (top) Three stages of the evolution of mRNA, translation machines, and genetic code. (**a**) Decoding of pre-mRNA by pre-tRNA/pre-aaRS machine resulting in the primitive GNC code. (**b**) Fecoding of short-chain mRNA by tRNA/aaRS machine in the transitional SNS code. (**c**) Decoding of long-chain mRNA by tRNA/aaRS/ribosome machine in the universal genetic code. Left column of the diagram shows the recruitment of amino acids during the evolution of the genetic code. (**B**) (bottom) Darwinian evolution began in the peptide/RNA world, an interplay between digital information and its supporting structure, such as a translation machine. The supporting structure is coupled to the information carrier by rules, such as RNA base-pairing and genetic code. The supporting structure is nourished by the chemicals and energy from the hydrothermal vent environment and provides the information carrier positive feedback.

In the early DIS stage, the pre-mRNA began to form with four codons (GGC, GCC, GAC, and GUC), which are assigned to four amino acids (valine, alanine, aspartic acid, and glycine). The primitive translation machine of pre-tRNA/pre-aaRS began to decode a short pre-mRNA strand creating a short chain of the biosynthetic protein. This is the first simple polypeptide chain made by DIS (Figure 18A and Figure 19A). In this stage, the primitive GNC code [55,56] appeared.

### 9.3. Decoding of Short-Chain mRNA by tRNA/aaRS Machine

**Figure 20:**
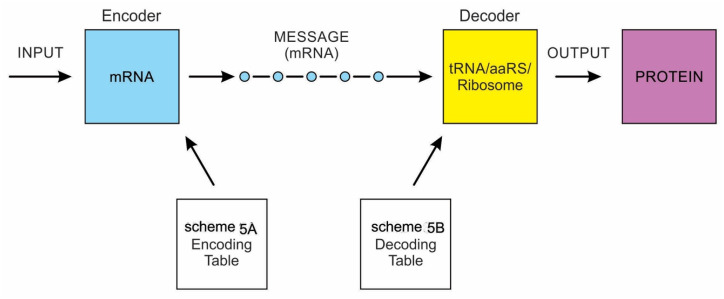
In a digital information transmission system, mRNA functions as the encoder of amino acid information and ribosome as a decoder of DIS to AIS to create protein.

In the next stage of DIS, pre-mRNA evolved into mRNA through gene duplication or linking several strands of pre-mRNA to increase storage capacity. mRNA recruited 16 codons (GGC, GGG, GCC, GCG, GAC, GAG, GUC, CUC, GUG, CCC, CCG, CAC, CAG, CGC, and CGG) or some combination of these codons to code 10 amino acids (valine, alanine, aspartic acid, glycine, glutamic acid, leucine, proline, histidine, glutamine, and arginine). Decoding of mRNA was performed by a tRNA/aaRS translation machine. These modifications of DIS and HIS gave rise to the SNS transitional code [50,51] (Figure 19B and Figure 20). The superior information-bearing qualities of mRNA, the excellent catalytic potential of aaRS, and the improved adaptor capacities of tRNA emerged with the gradual expansion of the genetic code. At this stage, tRNAs selected and recruited six additional amino acids. The expanded SNS code was refined through the symbiotic interactions of the tRNA/aaRS complex. The evolution of tRNA and aaRS considerably improved the translation system from the GNC to the SNS stage, but the code remains only moderately robust and susceptible to errors because of the limited redundancy.

### 9.4. Decoding of Long-Chain mRNA by tRNA/aaRS/ribosome Machine

**Scheme 4:**
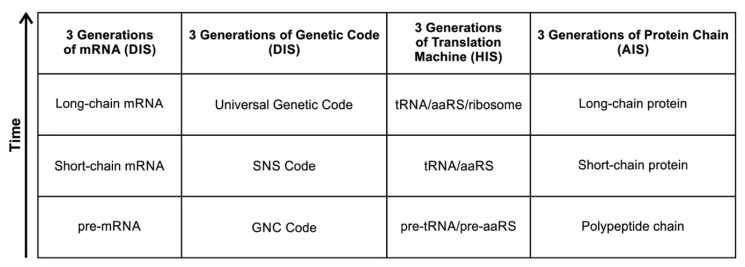
Three stages of the DIS, HIS, and AIS during the evolution of the genetic code. In GNC code, pre-mRNA was decoded by a pre-tRNA/pre-aaRS translation machine, creating a polypeptide chain. In SNS code, short-chain mRNA was decoded by a tRNA/aaRS machine, producing short-chain protein. In universal genetic code, long-chain mRNA was decoded by tRNA/aaRS/ribosome machine, manufacturing long-chain protein.

In the final stage of DIS, longer strains of mRNA evolved to store the recipe of complex proteins. Most likely, the first mRNA genes were short, no longer than 70 to 100 nucleotides (the modern genes run several thousand nucleotides), with the corresponding proteins [4]. In HIS, the translation machine was enhanced with the incorporation of the ribosome, an enormous hybrid of rRNAs and r-proteins. The ribosome improved the efficiency of translation, leading to the universal genetic code with its 20 amino acids and 64 codons (Figure 18C and Figure 19c; Scheme 4).

The peptide/RNA world entered the last stage in its evolution when translation had become sufficiently accurate to unambiguously link the sequences of individual proteins with the sequences of mRNA genes. This is the situation that exists today (with DNA carrying the primary genetic information), except those present-day systems are more accurate and refined through four billion years of evolution.

De Giulio [54] and Ikehara [55,56] modified the selection of amino acids proposed by Wong [51] to fit the three stages of the origin of the genetic code, as discussed in Section 7. Four amino acids that were available in the prebiotic soup (such as glycine (G), alanine (A), aspartic acid (D), and valine (V)) were selected during pre-mRNA encoding. Later, six more amino acids were recruited from the prebiotic environment (such as glutamic acid, leucine, proline, histidine, arginine, and glutamic acid) for encoding short-chain mRNA. These ten amino acids were precursors for forming the other ten amino acids along prebiotic pathways [54,55,56]. The more derived amino acids (such as isoleucine, methionine, threonine, asparagine, lysine, serine, phenylalanine, tyrosine, cysteine, and tryptophan) were used for encoding long-chain mRNA (Figure 19A). These twenty amino acids became the alphabet for proteins.

### 9.5. Flow of Information from Nucleic Acids to Proteins

The climax of the peptide/RNA world was the evolution of mRNA-directed protein synthesis via a translation machine. This was the first breakthrough in life to begin. Life depends on cycles of information between nucleic acids and proteins. In DIS, we see the progressive improvement of mRNA with more information content and the emergence of the genetic code. In HIS, there was a refinement of the translation machine. In AIS, twenty amino acids were generated for protein synthesis. Three prebiotic information systems, DIS, HIS, and AIS, worked in harmony for this remarkable achievement of making proteins (Scheme 4).

### 9.6. The Onset of Darwinian Evolution

Darwinian selection started very early in the origin of life and probably played a major role in abiogenesis. However, there is no consensus on when and how the Darwinian evolution emerged during abiogenesis. We suggested [40] that the emergence of the translation machines was the beginning of the Darwinian evolution, reciprocity between information and its supporting structure. This view was recently elaborated by Kunnev [6], who advocated the proposal that Darwinian evolution began when the information carrier molecules, such as mRNA, and its corresponding supporting structure, such as translation machines, began to interact, where information determines its supporting structure. The structural component is coupled to the information component through rules such as the Watson–Crick base-pair and the genetic code that maps changes in the information carrier to the changes in supporting structure. The structural component is nourished by environmental chemicals and energy that provide information carriers with positive feedback (Figure 19B). Kunnev proclaims that abiogenesis reached ’a point of no return’ at this digital stage to enhance life synthesis with the onset of Darwinian evolution.

### 9.7. Encoding and Decoding of Digital Information

**Scheme 5:**
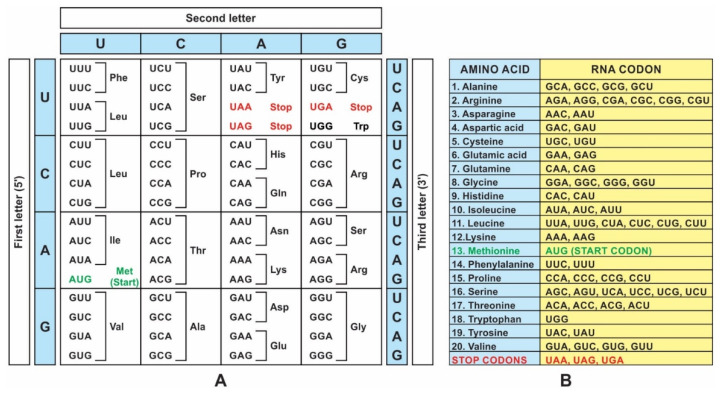
(**A**) Universal genetic code table shows 64 codons, each corresponding to a specific amino acid or stop signal. The start codon (AUG) is shown in green. Stop codons (UAA, UAG, and UGA) are shown in red. (**B**) In the genetic code, 20 amino acids are used in protein synthesis showing corresponding codons in redundancy.

The protein synthesis is the expected action of the encoding mRNA, an attribute of the digital information. A full digital communication system built upon the information from mRNA has emerged from our study with both an encoder and decoder, as shown in Figure 20. Initially, during the buildup of the digital information system (DIS), the original *coder of information* was pre-tRNA/tRNA that created codons by base-pairing and helped codon–amino acid association. The mRNA became the *transmitter* by linking codons, receiving the message, and encoding information of specific amino acids, and its strand became an *information channel*. Finally, tRNA/aaRS/ribosome became the *machine receiver* and *decoder* that obtained and processed the messages and performed the commanded action of linking amino acids into a polypeptide chain. No such components are apparent in a chemical system. Once the digital information system was established with the emergence of the universal genetic code, mRNA contained information programmed by natural selection; this information coded for the amino acid sequence of proteins. This protein would then perform specific functions within the protocell (Scheme 5).

## 10. Computer Information Systems vs. Prebiotic Information Systems

We discussed how three types of information systems, analog, hybrid, and digital, evolved one after another in the peptide/RNA world. Since these three systems of information are also present in human-made computers, it is instructive to make a comparison between life and computer–nature vs. human-made machines. A comparison between the biological systems and computer systems can enrich each discipline when fundamental knowledge becomes available. In both systems, distributed information processing operates without central control [62]. Of course, biological algorithms are designed by four billion years of natural selection and are more sophisticated and versatile than human-made algorithms. In this section, we clarify important differences between analog and digital systems and show some parallels in the evolution of biological and computer information systems.

Contrary to popular belief, the major difference between analog and digital computing is not its ability to process discrete versus continuous values but the internal structure of the computing system. The internal structure of a digital computing system is fixed, whereas the internal structure of an analog computing system is reconfigurable, and the analog computing system can change/reconfigure itself to deal with the given situation. Modern biological information systems evolved into the complex hybrid information system that takes advantage of both analog computing and digital computing. A hybrid information system is an integrated system of intercoupled analog and digital computing systems. A hybrid information system can be classified into three categories—analog-oriented, digital-oriented, or balanced-analog-digital. A balanced analog–digital–hybrid information system makes effective use of both types of components in an integrated form. An analog-oriented hybrid information system uses digital components mainly as auxiliary units.

Similarly, a digital-oriented hybrid information system uses analog components as auxiliary units. Initially, the biological information systems were predominantly analog in nature. These systems have evolved into complex, balanced hybrid systems. Even though much simpler than the natural biological systems, the evolution of human-made computer systems can be examined to shed some light on the evolution of biological information systems. Life has the same logical structure as a computer. Human-made computer systems have evolved from analog to digital to hybrid systems. We discussed below some examples of these three types of computer systems.

### 10.1. Analog Computer Systems

**Figure 21:**
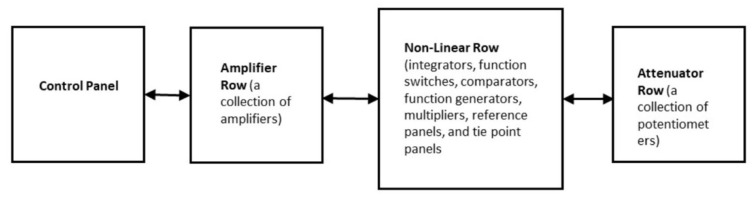
A Block diagram of the TR-10 analog computer built by Electronic Associates, Inc. EAI’s PACE TR-10, an electronic analog computer.

Human-made analog computer systems model a real-world problem using a set of differential equations. This mathematical model is then represented by an interconnection of various analog computing elements such as multipliers, summers, integrators, and amplifiers. The values are typically represented as continuous voltage or currents. Figure 21 shows a block diagram of EAI’s PACE TR-10 solid-state electronic analog computer built in 1961 (The EAI’s TR-10 Analog Computer [5]. A solution to a problem requires five steps using the PACE TR-10 computer (‘The EAI TR-10 Analog Computer’) [63]. These are (1) problem analysis, (2) block programming, (3) patching, (4) insertion of problem parameters, and (5) solution.

A thermometer is a simple analog computer. As the temperature varies, the mercury moves accordingly. Analog computers were dominant computer systems from the 1940s to about the mid-1970s [5]. They can easily be adapted to an analog world. They use continuous value representation, are much faster than a stored-program digital computer, and have less power. However, analog computers have very little memory, and they suffer more from noise and drift than digital computers. Continuous (analog) information is more sensitive to noise and challenging to restore than digital information encoded by codes. Due to noise, the output of an analog device cannot remain at the theoretically correct value for given inputs. The error usually tends to grow larger over time due to signal degradation and noise.

### 10.2. Digital Computer System

**Figure 22:**
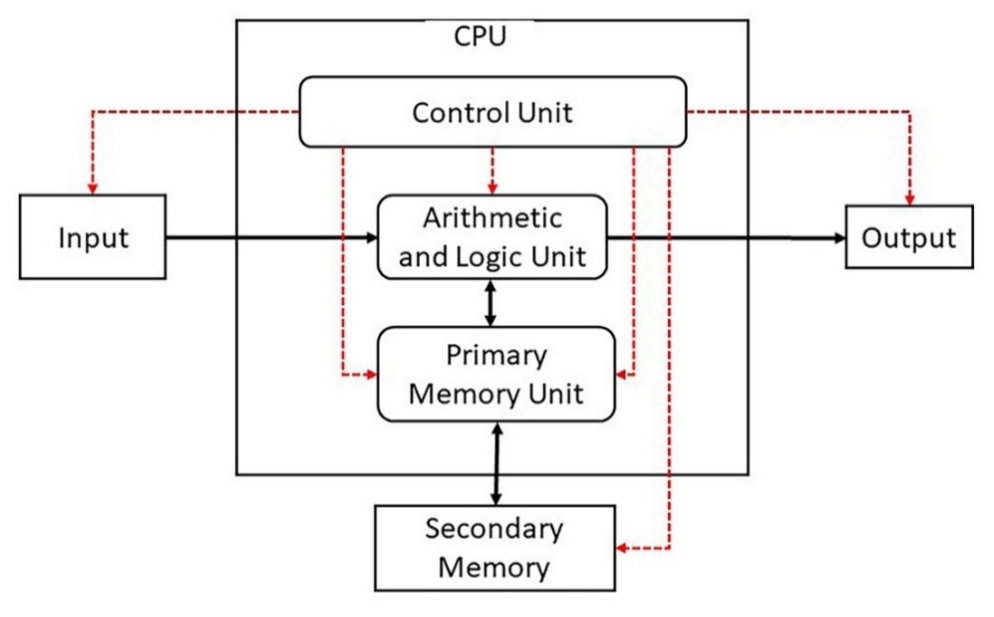
Block diagram of a basic digital computer with a uniprocessor CPU. Black lines indicate data flow, whereas red lines indicate control flow. Arrows indicate the direction of flow [adapted from Lambtron-owned work, CC BY-SA 4.0].

Unlike an analog computer system, a digital computer system has a fixed internal structure of its constituent computing elements and solves problems by executing a sequence of instructions read from memory. The instructions are based upon an algorithm [5]. Figure 22 shows the essential elements of a simple digital computer. These elements are fixed and are always interconnected in the same configuration. Digital computing is more versatile and accurate than analog computing. The transmission of digital information is less prone to signal fluctuation and signal degradation. The digital mode of data can be stored in a memory for a long time.

### 10.3. Hybrid Computer Systems

**Figure 23:**
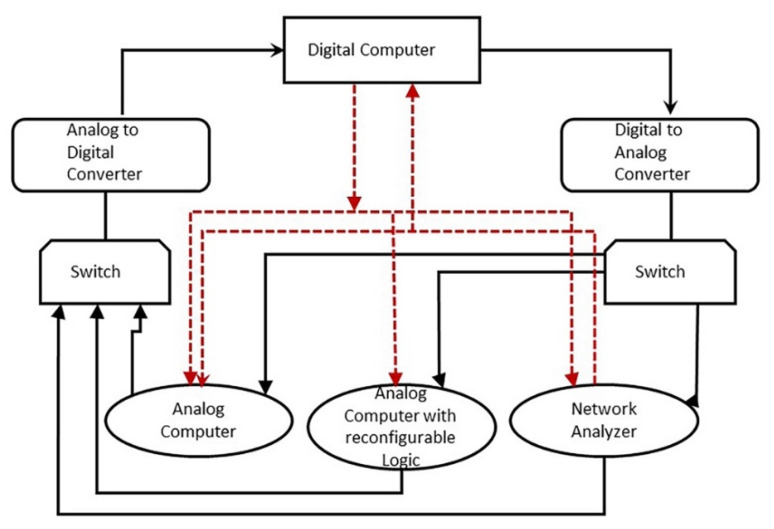
Block diagram of an available hybrid computer system.

A hybrid computer system integrates several kinds of computers that use different (analog and digital) representations of values and operates under a single control system. Basically, a hybrid computer system has the speed of analog computing and the accuracy of digital computing. Complex computing systems of today are hybrid computer systems. They can interact with their complex environment with actual time speed and accuracy. Figure 23 shows a block diagram of a general hybrid computer system. As shown, a Hybrid system has fixed and reconfigurable computing elements. Simple examples of a hybrid computer system are electrocardiogram machines and ultrasound machines. An electrocardiogram uses several sensors that pick up body signals and translate them into digital data. The controller then processes this digital data, and the output is usually generated in the form of a graph.

A more complex example of a hybrid computing system is NASA’s SpaceCube within the Raven module [64]. Raven and SpaceCube work together as the eyes and the brain to create the autopilot capability.

### 10.4. Coevolution of Prebiotic Information Systems and Biomolecules

**Figure 24:**
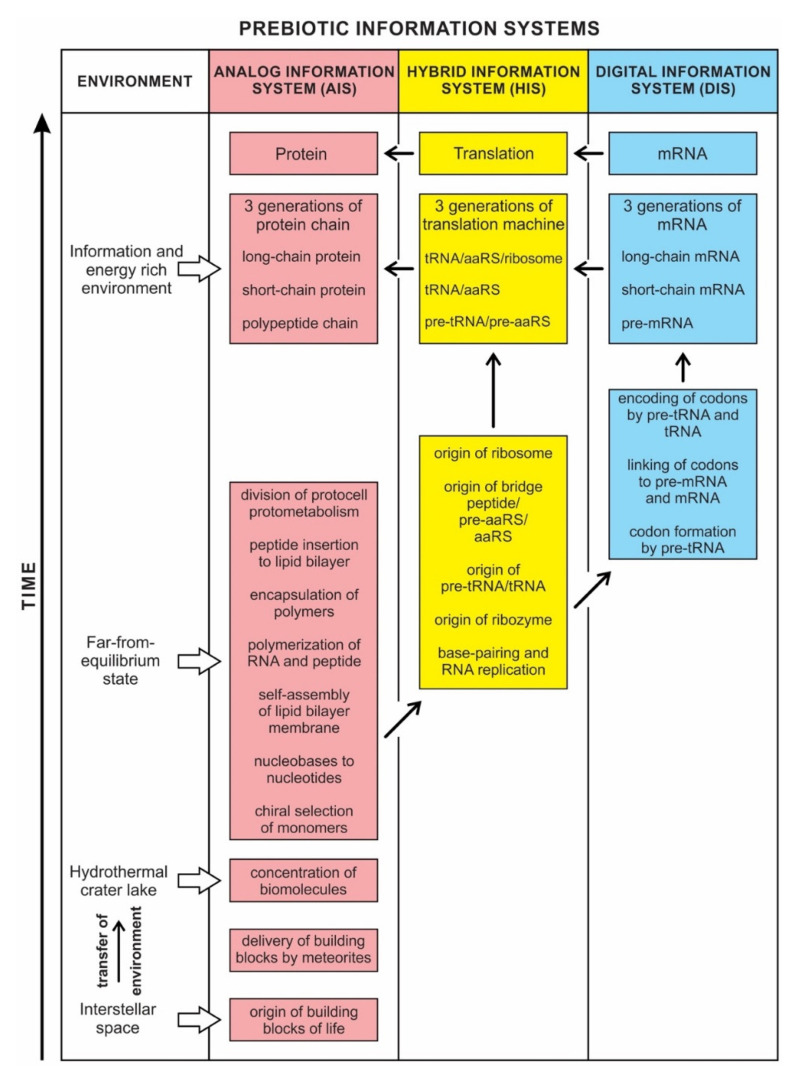
Coevolution of biomolecules with the biological information systems in the peptide/RNA world. An analog information system dominated the early stage of abiogenesis. With the emergence of nucleotides, hybrid information began to emerge. The origin of pre-mRNA and mRNA marked the digital revolution. During the origin of translation and the genetic code, the directionality of information flow from mRNA to proteins emerged.

Prebiotic information systems are more complex and more advanced computing systems than artificial computing systems. However, as discussed in the previous section, it was still insightful to see how human-made computing evolved, with changing computing requirements, from analog to digital and hybrid computing. It is remarkable to see the parallel between the evolution of human-made computing systems and biological information systems. Prebiotic information systems were predominantly analog in the beginning, and then building blocks of life advanced to hybrid computing with the advent of digital computing. We show the gradual evolution of biological information systems in the peptide/RNA world (Figure 24).

Unlike computers, it becomes clear from Figure 24 that analog information gave rise to digital information via hybrid information. Once the digital information is in place, the transfer of information from mRNA to proteins via a translation machine is established, but the reverse translation from proteins to mRNA is prohibited. Koonin [2] called this phenomenon the great biological principle—the irreversibility of digital to analog information. The digital information system and its unidirectional information flow are well-known. In the peptide/RNA world, the information flows from mRNA to proteins. Digital information always produces analog output.

## 11. Return to Analog Dynasty

Digital information paved the way for the second wave of analog information by manufacturing custom-made proteins according to the specificity of codon sequences of mRNA. Proteins were assembled from amino acids using information from the encoded mRNA. Emerging life did not ‘know’ that this interaction opened the way to one momentous development: RNA-dependent protein synthesis.

Different kinds of proteins control most of the functions of a cell, breaking down nutrients, assembling cellular components, copying DNA, and so on. They truly occupy a central position in the organization of the first cell. They act as enzymes that permit only a few of the many possible reactions among cellular components to take place. With their astonishing versatility, the protein enzymes catalyzed crucial biochemical reactions within protocells into more complex biomolecules in diverse metabolic pathways, whereas structural proteins provided strength and permeability in the cell membrane. They form channels in plasma membranes, allowing specific substances to enter and leave while excluding others. Protein molecules owe their properties to their three-dimensional shapes, which are determined by the amino acid sequences of their constituent chains. In turn, these properties determine how a protein biologically functions: whether it will bind specific organic molecules and catalyze their reactions or form a regular structure such as a helix and act as a building material. The only thing proteins cannot do is replicate themselves.

The first proteins were most likely short, about 25 amino acids long [4]. Protein molecules of this short length displayed enzyme-like activities. In contrast, many modern-day proteins contain several hundred amino acids. These long molecules likely arose by the gradual lengthening of mRNA sequences. Proteins are the primary functional biomolecules of life. Once formed, proteins perform many functions during biogenesis, including catalyzing metabolic reactions and reinforcing cell membranes. The overwhelming number of efficient proteins in the biochemical synthesis occurred in the protein/RNA world. These RNA-directed newly formed enzymes carried out hundreds of chemical reactions that took place in the protocell. Structural proteins, on the other hand, provide structure and support for the cell membrane. Once different proteins were synthesized, various mRNAs, ribosomes, and proteins began to accumulate in the primitive cytoplasm, which would be readily available when needed.

Proteins mediate most functions of modern cells. The newly synthesized protein enzymes helped catalyze and mediate these critical molecular evolutions, favored by strong selective forces. Three significant events followed in succession after the availability of template-directed proteins but with considerable overlap. These affected, first, the efficiency of the translation machinery, then, the resilience of the coding system, and finally, the quality of the synthesized proteins.

Some of the hierarchical stages of analog information in the protein world include (1) protein folding, (2) enzyme-substrate complex, (3) origin of phospholipid membrane, (4) origin of plasma membranes, and (5) chemiosmosis. Eventually, proteins gradually replaced ribozymes as the main biological catalysts. Since then, ribozymes have not taken on any major de novo catalytic function after the evolution of protein synthesis.

With the availability of protein, a new cycle of analog information began. A significant contribution of proteins in the prebiotic world is to create the ancient virus world. This ancestral virus is an amazing, sophisticated hybrid machine made of an mRNA genome inside a protein shell called a capsid. It replicates by infecting protocells by hijacking their translation machinery to create viral protein. The virus processes its digital information during replication to make its unique viral protein. In a separate paper, we will document how AIS in the protein world would give rise to HIS and the latter to DIS.

## 12. Discussion and Conclusions

Information is one of the key attributes of life, but the origin of prebiotic information systems remains a mystery. We propose transitional pathways from the cosmic building blocks of life to the complex prebiotic organic chemistry that led to the origin of information systems. The way the information flows through biomolecules has led to accelerating the chemical evolution and has provided directionality and complexity in abiogenesis. The molecular origin of life and its information systems are intimately linked in a complex feedback loop by a hierarchical organization. The way the information flows through biomolecules has led to accelerating the chemical evolution and has provided directionality and complexity in abiogenesis.

The scenario for the coevolution of biomolecules and information systems discussed in this paper, though purely hypothetical, depicts logical biochemical pathways in the peptide/RNA world. The predictions of our model can be tested by theoretical analysis or laboratory simulations as new evidence accumulates. The origin of life has produced organic molecules within a hierarchy of increasing size and complexity through time governed by information systems. How can a highly ordered living system emerge from a chaotic assemblage of space molecules in the hydrothermal crater vent environment? Most likely, prebiotic chemical processes were ruled by information systems that gave directionality and order. Information theory may shed new light on the origin of life. Life synthesis is an open system, and energy such as thioester and ATP must be fed in each step of improvement of information systems. Both information and energy in prebiotic synthesis originated from the environment. The new information paradigm discussed in this paper suggests that life processes three kinds of information—analog, hybrid, and digital—for streamlining molecular evolution. Life depends not only on the flow of energy but also on the flow of information. A living system stores information and processes and uses it to self-maintain and perpetuate itself. Living is an information processing system in which memory is maintained by analog, hybrid, and digital information.

Once the transitional information systems ratchet up the complexity ladder of hierarchy toward the first life, the stable memory of information sets in biomolecules permanently. The cosmic building blocks of life had embedded the Analog Information System (AIS) that became elaborated, modified, and fine-tuned during abiogenesis in the hydrothermal crater vent environment. Some examples of the hierarchical development of biomolecules and analog information systems built up slowly are manifested in the prebiotic synthesis. These are (1) concentration of cosmic biomolecule in the vent environment, (2) chiral selection of L-amino acids and D-ribose, (3) conversion of nucleobases to nucleotides, (4) self-assembly of lipid bilayer membrane, (5) polymerization of RNA and peptides to begin peptide/RNA world, (6) encapsulation of polymers, (7) peptide insertion to lipid bilayer, (8) protometabolism, and (9) division of protocells.

As analog information created versatile, noncoding RNA molecules, it paved the way for the emergence of the Hybrid Information System (HIS). HIS created the components of the translation machine. Some of the significant steps in hybrid information along with molecular evolution include (1) base-pairing and RNA replication, (2) origin of ribozyme, (3) origin of pre-tRNA/tRNA, (4) origin of bridge peptides/pre-aaRS/aaRS, and (5) origin of the ribosome.

The HIS led to the Digital Information System (DIS). It occurred late in abiogenesis. We proposed a new model for the origin of genes before transcription in the peptide/RNA world. We developed our model of assembling and encoding mRNAs by tRNAs based upon several assumptions and evolutionary principles. The pre-tRNA molecules designed and created custom-made codons for storing amino acid information in three steps: (1) codon formation by pre-tRNA and tRNA, (2) linking of codons to pre-mRNA and mRNA, and (3) encoding of codons by pre-tRNAs and tRNAs. DIS is encoded in the linear sequences of nucleotides in pre-mRNA and mRNA. Codon–amino acid associations create genetic memory that resides in the codon sequences of mRNA for translation and the genetic code. The encoded mRNA molecules represent the ancestral genes before the advent of DNA.

The central dogma of molecular biology states that ‘DNA makes RNA makes proteins’. We are interested in the possibility of intercepting the second step in the peptide/RNA world: ‘RNA makes proteins’. Two different RNA molecules, tRNA and mRNA, are required in this step for translation. Our new view of *prebiotic dogma* of molecular biology states, ‘tRNA makes mRNA makes proteins’.

By using a computer simulation and visualization model of the possible pathways for the origin and encoding of codons in pre-mRNA and mRNA by pre-tRNA and tRNA, we showed the step-by-step evolution of the encoding of mRNA by charged tRNA molecules. During this process, genetic code evolved as a memory bank for codon–amino acid mapping.

The evolution of the genetic code in the early peptide/RNA world is dependent on steadily improving the translation machinery for codons, anticodons, tRNAs, and amino acids. It developed in three stages: (1) GNC code, where pre-mRNA used pre-tRNA/pre-aaRS translation machine to generate short polypeptide chain; (2) SNS code, where short-chain mRNA utilized tRNA/aaRS translation machine to produce short-chain protein; and (3) in universal genetic code, where long-chain mRNA used tRNA/aaRS/ribosome machine to create long-chain protein. The genetic code is an abstract, immaterial, and nonphysical set of rules stored in the memory bankand is universal in all life. That the code is universal is extremely significant, for it suggests that it was used by the Last Universal Common Ancestor (LUCA) and is robust enough to have survived four billion years. Life does not reinvent the code when a new species appears. It must be deeply embedded in its nucleotides and translation machines. Digital information becomes unidirectional, where information flows from DIS to HIS to AIS during protein synthesis. Digital information always produces analog output (such as mRNA --> proteins), but the reverse information flow from analog to digital translation is forbidden. With the advent of proteins, a new information cycle begins with AIS, which would give to HIS, the latter to DIS. As we understand the mechanism of information systems in primitive cells, it will have broader applications in modern technology because molecular memory could be the future of data storage devices.

## Figures and Tables

**Figure A1:**
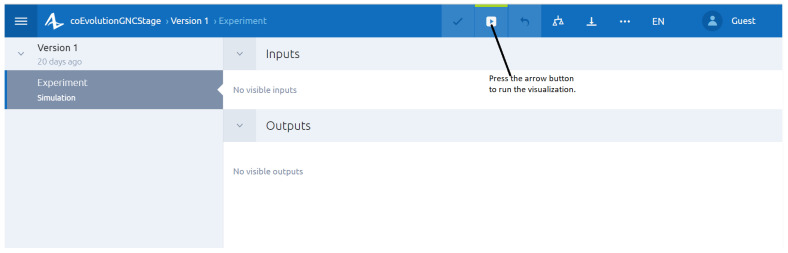
A screenshot of the webpage showing the button to be pushed.

**Figure A2:**
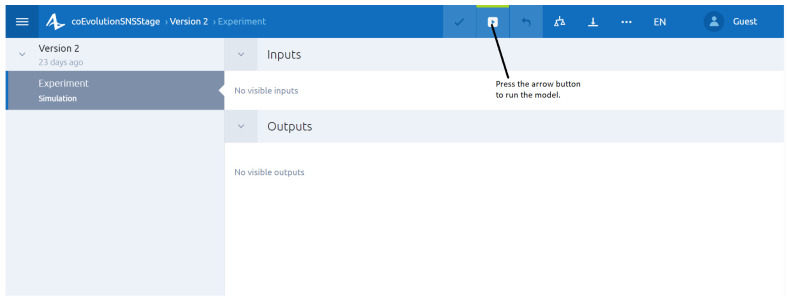
A screenshot of the webpage showing the button to be pushed.

**Figure A3:**
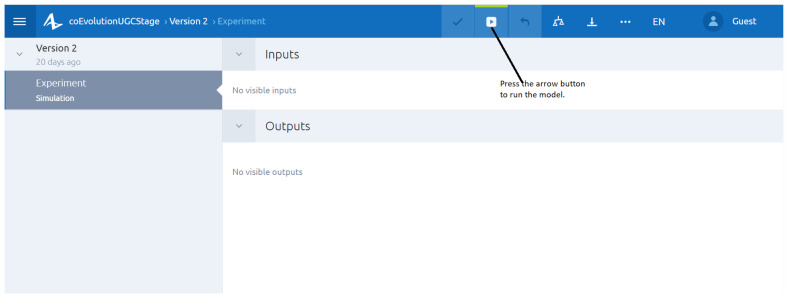
A screenshot of the webpage showing the button to be pushed.

## Data Availability

Not applicable.

## Appendix A

This appendix describes how to run the visualization model developed in the AnyLogic software for encoding codons in each stage of the genetic code. The visualization models are hosted on the AnyLogic Cloud, available online on the internet, and can be run under a browser such as Chrome, Internet Explorer, Fire Fox, etc.

### Appendix A.1. Instructions for Stage I Visualization—GNC Stage

Please follow these steps to visualize the encoding process of four codons by charged pre-tRNA molecules in the GNC code:

Step 1. Paste the following URL link in your browser’s address line and go to that link: https://cloud.anylogic.com/model/deada2dd-97b7-43e4-8985-e44abca2a6f8?mode=DASHBOARD&experiment=9e487ce5-3a6b-40c5-870d-a63264e3640b (accessed on 1 February 2022).

Step 2. You should see a web page that looks like the one shown in Figure A1.

Step 3. Press the button as indicated in Figure A1. The visualization model should start running in a few seconds.

### Appendix A.2. Instructions for Stage II Visualization—SNS Stage

Please follow these steps to visualize the encoding process of 10 codons (4 + 6) with six new codons by charged tRNA molecules in the SNS code:

Step 1. Paste the following URL link in your browser’s address line and go to that link: https://cloud.anylogic.com/model/cce27d60-e6b4-4633-be88-cabf062bc2a8?mode=DASHBOARD&experiment=acdc89a8-678f-42dc-83ec-cee847a81125 (accessed on 1 February 2022).

Step 2. You should see a web page that looks like the one shown in Figure A2.

Step 3. Press the button as indicated in Figure A2. The visualization model should start running in a few seconds.

### Appendix A.3. Instructions for Stage III Visualization—UGC Stage

Please follow these steps to visualize the encoding process of 61 codons (16 + 45) with 45 new codons by charged tRNA molecules in the UGC stage model:

Step 1. Paste the following URL link in your browser’s address line and go to that link: https://cloud.anylogic.com/model/a183f773-bfa2-4e0a-bda8-66b034bd7d86?mode=DASHBOARD&experiment=6205ea99-a3fd-4ad5-8c71-482c45343bb8 (accessed on 1 February 2022).

Step 2. You should see a web page that looks like the one shown in Figure A3.

Step 3. Press the button as indicated in Figure A3. The visualization model should start running in a few seconds.
